# Descriptions of two new genera of the spider family Caponiidae (Arachnida, Araneae) and an update of *Tisentnops* and *Taintnops* from Brazil and Chile

**DOI:** 10.3897/zookeys.622.8682

**Published:** 2016-10-06

**Authors:** Antonio D. Brescovit, Alexander Sánchez-Ruiz

**Affiliations:** 1 Laboratório Especial de Coleções Zoológicas, Instituto Butantan, Av. Vital Brasil, 1500, Butantã, São Paulo, São Paulo, Brazil, 05503-900

**Keywords:** Taxonomy, new species, haplogynae, new genus, Neotropical region

## Abstract

New members of the spider family Caponiidae from Brazil and Chile are presented. Three new species in previously known genera are described: *Taintnops paposo* **sp. n.** from Chile, and the Brazilian *Tisentnops mineiro* **sp. n.** and *Tisentnops onix* **sp. n.**, both belonging to a genus known only from its damaged type. Additionally, two new non–nopine Brazilian genera are proposed: *Nasutonops* **gen. n.** including three new species: *Nasutonops chapeu* **sp. n.**, *Nasutonops sincora* **sp. n.** and *Nasutonops xaxado* **sp. n.**; and *Carajas* **gen. n.**, known only from the type species *Carajas paraua* **sp. n.** Both new genera have entire, rather than sub-segmented tarsi. Therefore, they are not included in the caponiid subfamily Nopinae. *Nasutonops* **gen. n.** is characterized by the presence of a projected clypeal horn, unique among caponiids. Additionally, the first blind caponiids are described: *Tisentnops mineiro* **sp. n.** from the state of Minas Gerais and *Carajas paraua* **sp. n.** from the state of Pará. Both of these species are found only in caves and completely lack eyes.

## Introduction

The family Caponiidae is currently represented by 15 genera and 98 species (World Spider Catalog 2016). The family was divided into two subfamilies by Petrunkevitch (1939): Nopinae includes genera with sub-segmented tarsi and Caponinae includes genera with entire tarsi. However, based on current data, only Nopinae could be monophyletic (Platnick 1995; Platnick and Lise 2007), whereas those genera with entire tarsi (non-nopine) may form a basal and paraphyletic group (Platnick 1994a; Kranz-Baltensperger et al. 2009). Although some of these genera seem to be more closely related each other than with the Nopinae (Platnick and Jäger 2008).

Currently nine non-nopine genera are known: *Caponia* Simon, *Diploglena* Purcell, *Iraponia* Kranz-Baltensperger, Platnick & Dupérré and *Laoponia* Platnick & Jäger from Africa and Asia, and *Calponia* Platnick, *Caponina* Simon, *Notnops* Platnick, *Taintnops* Platnick and *Tisentnops* Platnick from the New World. Recently, the first genera from Asia were described (Platnick and Jäger 2008; Kranz-Baltensperger et al. 2009), and the African genus *Diploglena* was revised (Haddad 2015). However, *Caponia*, the first described Caponiidae genus, has not been studied since the works of Purcell (1904) and Lessert (1936) in the early twentieth century. Other than *Caponina*, the non–nopine New World genera are monotypic, and *Tisentnops* is known only from an extremely damaged type species, precluding a detailed morphological description (Platnick 1994b).

During several field trips to collect haplogynae spiders in Brazil and Chile, three new species of the genera *Taintnops* and *Tisentnops* were found (the Chilean *Taintnops paposo* sp. n. and the Brazilian *Tisentnops mineiro* sp. n. and *Tisentnops onix* sp. n.). The new species allowed us to gather detailed morphological information, expand the distributional range, and provide information about the natural history of these two genera. Additionally, we found two new non–nopine Brazilian genera: *Nasutonops* gen. n. and *Carajas* gen. n.; the first with three new species *Nasutonops chapeu* sp. n., *Nasutonops sincora* sp. n., *Nasutonops xaxado* sp. n., and the second known from only one species *Carajas paraua* sp. n. All these new genera and species are also described in this paper.

Some of the new species are remarkable among caponiids. We describe the first known blind caponiids: *Tisentnops mineiro* sp. n. and *Carajas paraua* sp. n.; both species are known only from caves and completely lack eyes. Another unique characteristic occurs in the new genus *Nasutonops*, which has a hard, distally projected clypeal horn, similar to the goblin spider genus *Unicorn* Platnick & Brescovit (see Platnick and Brescovit 1995: figs 1–2). No other caponiids are known to have such a dramatic carapace modification.

## Material and methods

Morphological observations and illustrations were made using a Leica MZ12 stereomicroscope with a camera lucida. Photographs were taken with a Leica DFC 500 digital camera mounted on a Leica MZ 16A stereomicroscope. Extended focal range images were composed with Leica Application Suite version 2.5.0. The female internal genitalia were dissected following Levi (1965), and soft tissues were digested after immersion in clove oil for visualization of internal structures. SEM images were taken under high vacuum in a FEI Quanta 250 Scanning Electron Microscope from the Laboratório de Biologia Celular do Instituto Butantan and in a LEO 1450VP from the Laboratório de Microscopia Eletrônica do Museu Paraense Emílio Goeldi (MPEG). All figures were edited using Adobe Photoshop CS5 ver. 12.0. Descriptions and measurements follow Platnick (1994b). Measurements are in millimeters (mm) and were made using an ocular micrometer.

The specimens examined are deposited in the following collections (Abbreviation and curator in parentheses): American Museum of Natural History, New York (AMNH, N.I. Platnick); Instituto Butantan, São Paulo (IBSP, A.D. Brescovit); Coleção de Invertebrados Subterrâneos da Universidade Federal de Lavras, Lavras (ISLA, R.L. Ferreira), Museu Paraense Emílio Goeldi, Belém (MPEG, A.B. Bonaldo).

## Taxonomy

### Tisentnops

Taxon classificationAnimaliaAraneaeCaponiidae

Platnick, 1994

Tisentnops Platnick, 1994b: 9 (Type species by original designation Caponina leopoldi Zapfe).

#### Note.

**Figure 1.**
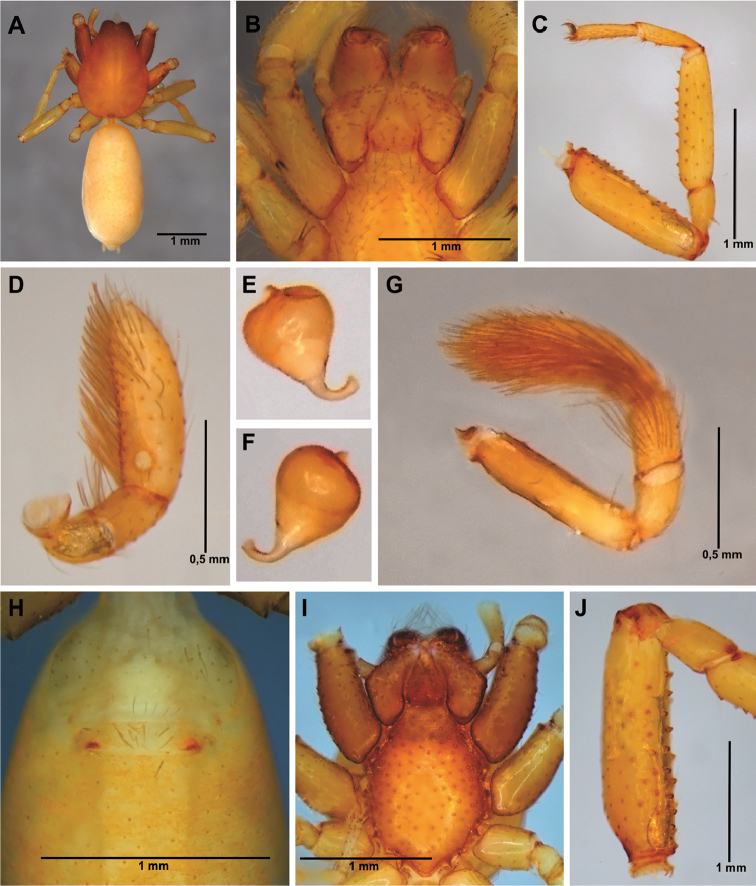
*Tisentnops mineiro* sp. n., male holotype (**A–G, J**), female paratype, IBSP 191294 (**H–I**). **A** habitus, dorsal view **B** mouthparts, ventral view **C** leg I, prolateral view **D** left male palp, ventral view, showing alveolus (bulb removed) **E** male bulb, prolateral view **F** same, retrolateral view **G** left male palp, prolateral view (bulb removed) **H** female external genitalia, ventral view **I** carapace, ventral view **J** male femur I, retrolateral view.

Platnick (1994b: 9) reported that the holotype of *Tisentnops leopoldi* (Zapfe) was in the Museo de Historia Natural de Santiago de Chile, and this specimen, probably a female, was extremely deteriorated. The type was examined by the first author during a recent trip to Chile. Now, with specimens of *Tisentnops mineiro* sp. n. and *Tisentnops onix* sp. n., an emendation is presented to the generic description. The eyes previously used in Platnick (1994b) as diagnostic characters are no longer applicable because some species in this genus lack eyes (Figs 1A, 5E).

#### Diagnosis.

**Figure 2.**
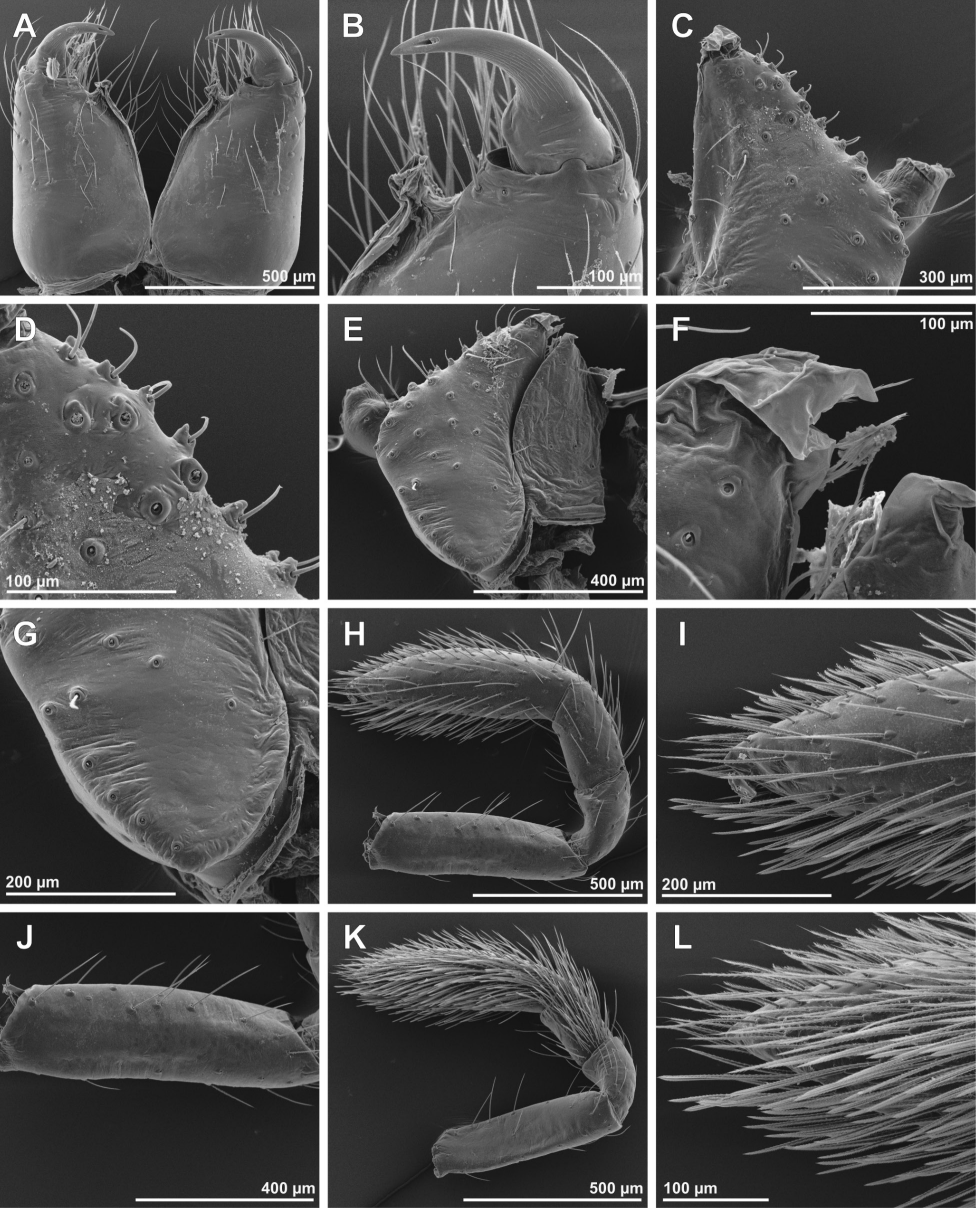
SEM images of *Tisentnops mineiro* sp. n., female (IBSP 191297) (**A**–**L**). **A**–**B** chelicerae, ventral view **C** left endite, ventral view **D** detail on left endite, ventral view **E** right endite and labium, ventral view **F** detail apical on right endite, ventral view **G** base of right endite, ventral view **H** left pedipalp, retrolateral view **I** tip detail of left pedipalp, retrolateral view **J** femur of left pedipalp, retrolateral view **K** right pedipalp, prolateral view **L** tip detail of right pedipalp, prolateral view.

Members of the genus can be easily separated from all other caponiid genera by the distally widened palpal endites, as in *Diploglena*, but uniquely modified with a series of setae with elongated sockets in the sub-marginal and anterior margin(Fig. 2C–E). These elongated sockets may also be present in anterior legs (Fig. 1C, J).

#### Description.

**Figure 3.**
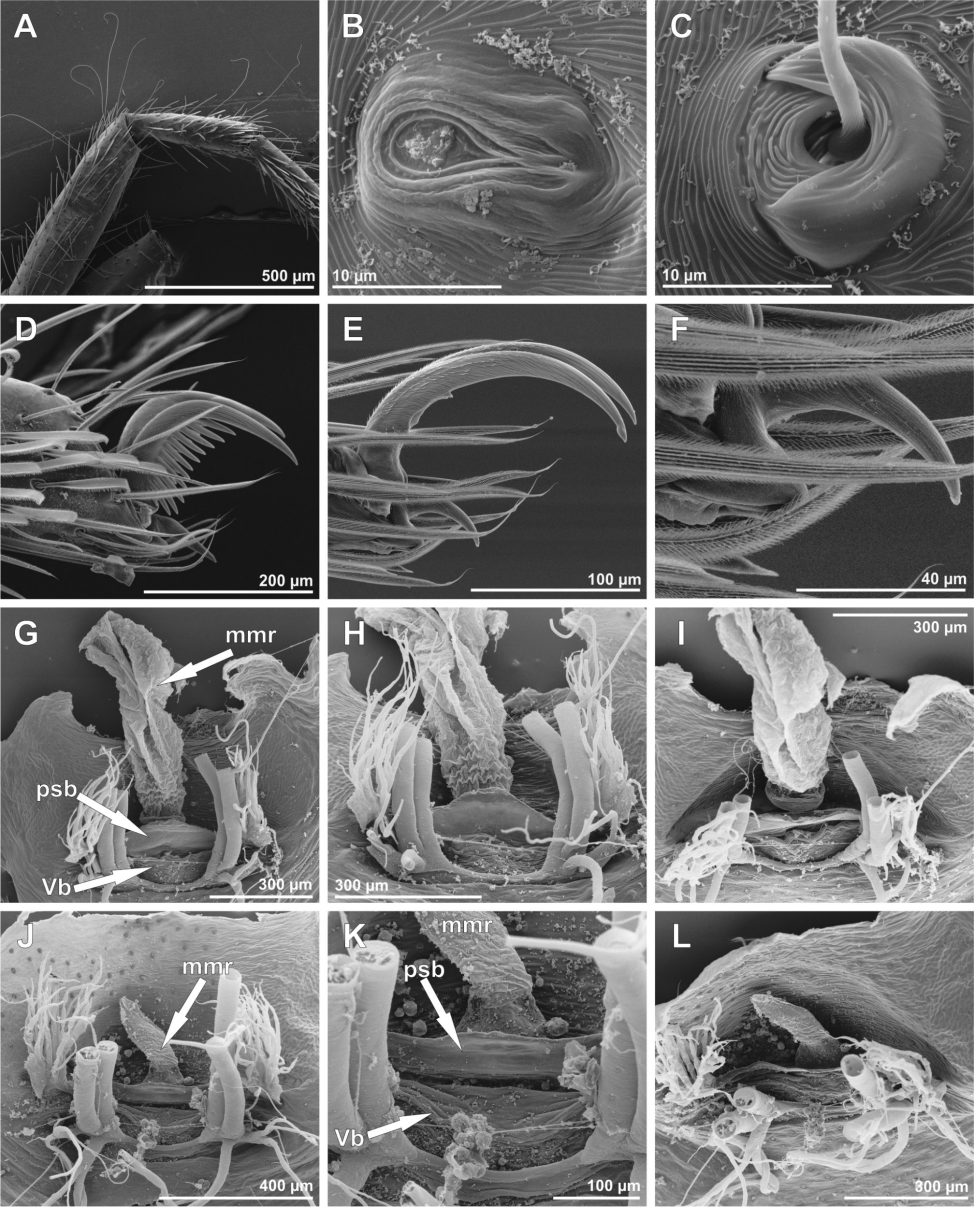
SEM images of *Tisentnops mineiro* sp. n., female (IBSP 191301) (**A**–**I**), female (IBSP 191297) (**J–L**). **A** right tibia, metatarsus and tarsus I, prolateral view **B** tarsal organ on leg I, dorsal view **C** tricobothrial base on leg I, dorsal view **D** claws on left tarsus I, prolateral view **E** claws on left tarsus IV, prolateral view **F** same, detail unpaired claw, prolateral view **G** female internal genitalia, dorsal view **H** same, posterior view **I** same anterior view **J** same, dorsal view **K** same, detail of receptaculum, dorsal view **L** same, anterior view (mmr = membranous anteromedian receptaculum; psb = pair of sclerotized bars; Vb = V-shaped bar).

**Figure 4.**
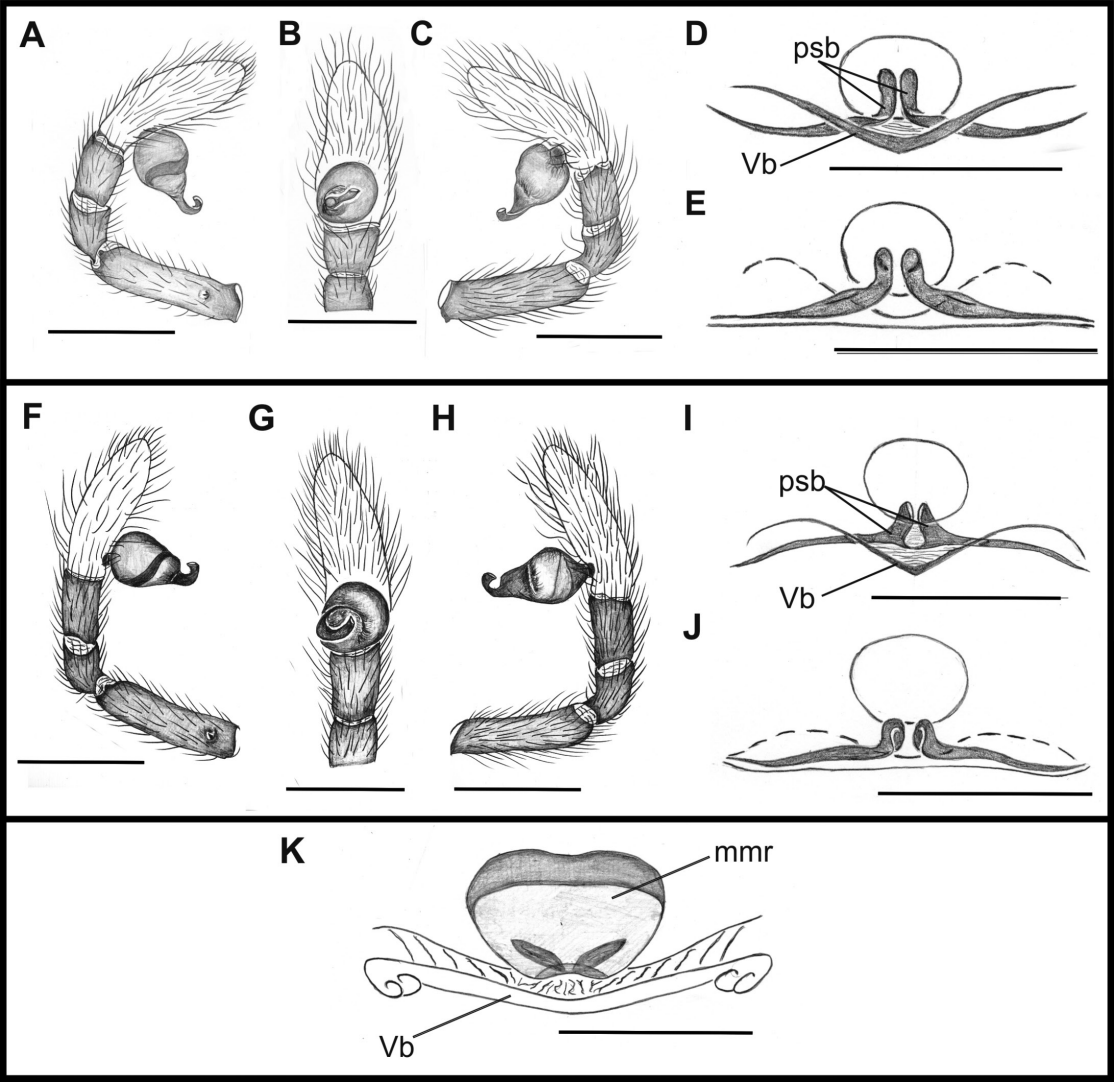
*Tisentnops mineiro* sp. n., male from Moeda, Minas Gerais (**A–C**), female from Santa Bárbara, Minas Gerais (IBSP 191313) (**D–E**), *Tisentnops onix* sp. n., male holotype **(F–H)**, female paratype (**I–J**). *Taintnops paposo* sp. n. from Taltal, Antofagasta, Chile (**K**). **A** left pedipalp, prolateral view **B** same, ventral view **C** same, retrolateral view **D** sclerotized parts of internal genitalia, ventral view **E** same, dorsal view **F** left pedipalp, prolateral view **G** same, ventral view **H** same, retrolateral view **I** sclerotized parts of internal genitalia, ventral view **J** same, dorsal view **K** internal genitalia, ventral view (mmr = membranous anteromedian receptaculum; psb = pair of sclerotized bars; Vb = V-shaped bar). Scale bars: **A–C, F–H** = 1 mm, **D–E, I–K** = 0.5 mm.

**Figure 5.**
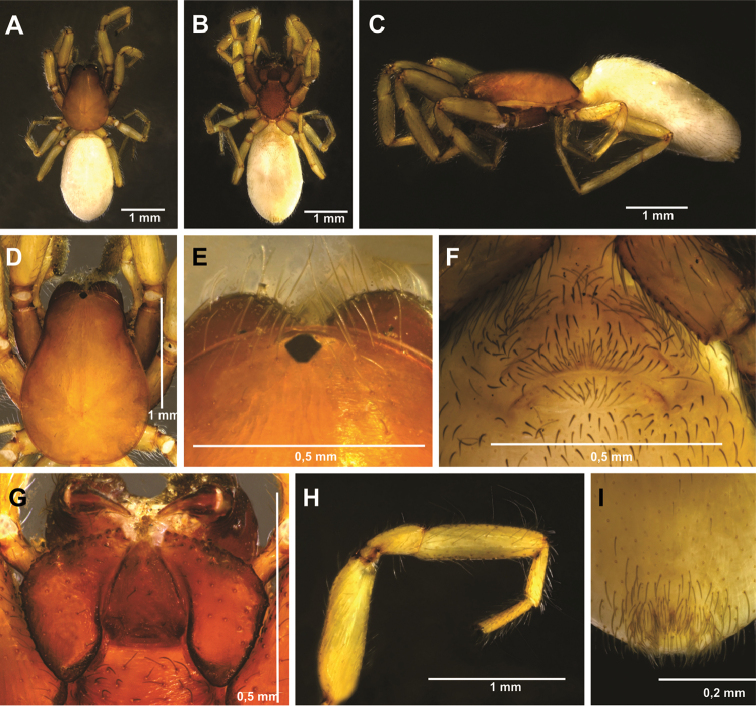
*Tisentnops onix* sp. n., female holotype (**A–I**). **A** habitus, dorsal view **B** same, ventral view **C** same, lateral view **D** carapace, dorsal view **E** ocular area, dorsal view **F** abdomen, genital area, ventral view **G** mouthparts, ventral view **H** leg I, prolateral view, showing long hairs **I** spinnerets, ventral view.

Described by Platnick (1994b), but new data are included here. Caponiids of moderate-size (Figs 1A; 17A, C–D), eyeless (Figs 1A; 17A), or with two small eyes, near the anterior border of the carapace (Fig. 5E). Carapace broadly oval, anteriorly narrowed to less than half its maximum width, pars cephalica rounded, ocular tubercle not projecting forward; pars thoracica relatively flat, gradually sloping toward laterally and posteriorly, without submarginal elevations opposite the coxal bases or submarginal depressions opposite the coxal interspaces (Fig. 5C); cuticle smooth; clypeus unremarkable (Fig. 5E); thoracic groove obsolete (Figs 1A, 5D). Cheliceral paturon with long, relatively strong bristles; short fang with unmodified base (Fig. 2B); median lamina long with short tooth-shaped tip and a white membranous lobe opposite the tip of the cheliceral fang (Fig. 2A–B); lateral surface with short stridulatory ridges, pick on prolateral side of palpal femur, next to base of male palp (Fig. 4A, F) and inconspicuous on the female pedipalp (Fig. 2K). Endites convergent, distally widened and extending far beyond the posterior margin of the labium (Fig. 2E, G), not touching at tip, covered by setae with elongated sockets which form a row along the anterior margin (Figs 1B, I; 2C–E; 5G), serrula absent. Labium almost pentagonal, much longer than wide, fused to sternum (Figs 1B, I; 5G), slightly invaginated at base, covered with a few scattered setae, distal area acuminate (Fig. 2E–F); labrum short, narrow, with few setae. Sternum longer than wide, covered with scattered large setae, without radial furrows between coxae, not fused to carapace (Fig. 1I); cephalothoracic membranes without epimeric sclerites, but short triangular sclerites extend from sternum between coxae I and II, II and III, and III and IV; shorter triangles extend to coxae II-IV. Leg formula 4123; legs without spines, legs I and II with setae with elongated sockets (Fig. 1C, J); metatarsi and tarsi entire, without sub-segmentation or membranous processes; tarsi with three claws; paired claws with approximately ten teeth on legs I-II, distal teeth largest (Fig. 3D); legs III-IV with paired claws very long, with two small basal teeth, distal teeth largest (Fig. 3E); unpaired claw shorter than paired ones on all legs, without teeth (Fig. 3D–F). Tibiae, metatarsi, and tarsi with long trichobothria in a single row (Figs 3A, 5H), bases with semicircular rim bearing low ridges (Fig. 3C); tarsal organ exposed, with very short longitudinal ridge in proximal end, covered by semicircular long ridges (Fig. 3B); female palpal tarsus moderately elongate, prolateral surface densely covered with setae, retrolateral surface covered by few setae (Fig. 2H, K). Abdomen with only slightly sclerotized epigastric area, with two pairs of respiratory spiracles; posterior spiracles connected by rebordered groove extending farther back at middle than at sides (Fig. 1H). Six spinnerets (Fig. 5I) in typical caponiid arrangement (Platnick et al. 1991: 56, Sánchez-Ruiz et al. 2010: 96, 140). Male palpal patella and tibia short, unmodified; cymbium ovoid, circular alveolus, prolateral surface densely covered with strong setae; bulb stout and globose; cylindrical embolus, slightly curved with enlarged tip, directed retrolaterally (Figs 1D–G; 4A–C, F–H). External female genitalia without scutum, weakly sclerotized (Figs 1H; 5F). Internal female genitalia with an elongate anteromedian membranous receptaculum accompanied by wide, transverse and anteriorly directed sclerotized bars that are coated with a transparent hyaline membrane, and by a V-shaped dorsal fold internally on the posterior plate (Figs 3G–L, 4D).

#### Distribution.

Known from Chile and southeastern Brazil.

### Tisentnops mineiro sp. n.

Taxon classificationAnimaliaAraneaeCaponiidae

http://zoobank.org/B7485A08-D81A-4EB7-B350-7B1DD7F07304

Figures 1 , 2 , 3 , 4A–E , 17A

#### Types.

Male holotype from Gruta da Serra da Moeda (20°19'58"S, 44°03'10"W), Moeda, Minas Gerais, Brazil, X.2005, R.L. Ferreira, deposited in IBSP 191293; female paratype from Gruta MP-10 (20°15'58"S, 43°53'16"W), Itabirito, Minas Gerais, Brazil, IX.2007, R.L. Ferreira, deposited in IBSP 191294.

#### Etymology.

The specific name is the designation for the native people from the state of Minas Gerais, Brazil, where this species was found.

#### Diagnosis.

*Tisentnops mineiro* sp. n. is easily separated from other species of the genus by the absence of eyes (Figs 1A, 17A).

#### Description.

**Male (holotype).** Total length 3.6. Carapace 1.6 long, 1.2 wide. Coloration: cephalothorax orange reddish. Palps yellow. Legs yellow, except coxae I-II orange-reddish. Abdomen uniformly yellow-white. Eyes absent of setae with elongated bases forming a row on the anterior margin of the endites, infrequent on the sub-marginal part (Fig. 1B). Leg measurements: I: femur 1.3/ patella 0.6/ tibia 1.1/ metatarsus 0.6/ tarsus 0.6/ total 3.7; II: 1.1/ 0.6/ 1.0/ 0.5/ 0.4/ 3.6; III: 0.9/ 0.4/ 0.6/ 0.6/ 0.4/ 2.9; IV: 1.1/ 0.6/ 1.1/ 0.8/ 0.7/ 4.3. Sockets bases of setae elongate on leg I forming an asymmetric row on the ventral margin of the femur and the tibia (Figs 1C, J). Palpal cymbium twice the length of the bulb, embolus short and curved, half the length of the bulb (Figs 1D–F; 4A–C).

**Female (paratype)**. Total length 3.8. Carapace 1.7 long, 1.2 wide. Coloration: cephalothorax and legs orange, except legs III-IV yellow and external border of endites brown. Abdomen grayish. Palpal endites as in male, but with fewer sub-marginal modified sochets (Fig. 1I). Leg measurements: I: femur 1.1/ patella 0.5/ tibia 0.9/ metatarsus 0.5/ tarsus 0.4/ total 3.4; II: 1.0/ 0.5/ 0.7/ 0.5/ 0.4/ 3.1; III: 0.8/ 0.4/ 0.5/ 0.6/ 0.5/ 2.8; IV: 1.2/ 0.5/ 0.9/ 0.8/ 0.5/ 3.9. Internal female genitalia with a triangular anterior margin of the hyaline membrane that covers transverse sclerotized bars, an elongate membranous anteromedian receptaculum with a wide base, and a transverse, enlarged, V-shaped dorsal fold (Figs 3G–L, 4D–E).

#### Other material examined.

BRAZIL, **Minas Gerais**: Conceição do Mato Dentro, Cave CSS-05 (18°55'02"S, 43°25'41"W), 1♀, 12–26/VIII/2013, L. Madeira (IBSP 191297, MEV); Cave CSS-06 (18°55'02"S, 43°25'42"W), 1♀ 2 imm., 15/XII/2010-14/I/2011, L. Tunes (IBSP 191299); Cave CSS-06 (18°55'02"S, 43°25'42"W), 1 imm., 15/XII/2010-14/I/2011, C.R.A. Souza (IBSP 191300); Cave CSS-06 (18°55'02"S, 43°25'42"W), 1♀, 03-13/V/2011, K. Pinheiro (IBSP 191301, MEV); Cave SERP-118 (19°05'55"S, 43°20'34"W), 2♀, 03/XII/2013, L.G.S. Soares (IBSP 191304); Santa Bárbara, Cave SG-07 (20°02'59"S, 43°41'05"W), 1 imm., 26-30/IX/2011, K. Pinheiro (IBSP 191312); Cave SG-10 (20°03'18"S, 43°41'09"W), 2♀, 26-30/IX/2011, K. Pinheiro (IBSP 191313); Cave AP-47 (20°01'40"S, 43°40'53"W), 1♀, 31/IV-05/V-2012, G.P. Perroni (IBSP 191314), Cave AP-38 (20°01'51"S, 43°40'45"W); 1♀, 31/IV-05/V-2012, G.P. Perroni (IBSP 191317); Nova Lima, Serra da Piedade, Cave SC-11 (19°57'03"S, 43°53'28"W), 2♀ 4 imm., 18/VII- 21/XI/2014, M. P.A. Oliveira (IBSP 191296, IBSP 191298); Cave SC-07 (19°57'39"S, 43°53'28"W), 1 imm., 18/XI/2014, M.P.A. Oliveira (IBSP 191302); Cave SC-05 (19°57'05"S, 43°53'28"W), 1 imm., 21/VII/2014, M.P.A. Oliveira col. (IBSP 191303); Rio Acima, Cave VG-27 (20°06'59"S, 43°53'54"W), 1♀, 02-10/VIII/2011, I. Cizauskas et al. (IBSP 191318); Cave VG-28 (20°06'58"S, 43°53'55"W), 1 imm., 02-10/VIII/2011, I. Cizauskas et al. (IBSP 191319); Caeté, Gruta do Triangulo (19°49'03"S, 43°40'51"W), 2♀ 1 imm., 25/III/2012, M.E. Bichuette & J.E. Gallão (IBSP 191322-191323); Cave AVG-30 (19°49'21"S, 43°41'50"W), 1♀, 18/V/2013 (IBSP 191324); Cave AVG-47 (19°49'22"S, 43°41'44"W), 3♀ 6 imm., 19/XII/2012-17/V/2013 (IBSP 191325-191326); Cave AVG-66 (19°49'28"S, 43°41'34"W), 1♂, 08/IV/2014 (IBSP 191305), all collected by M. P.A. Oliveira; Itabirito, Gruta MP-10 (20°15'58"S, 43°53'16"W), 1 imm., 30/III/2012, Equipe Carste col. (IBSP 191295); Cave VL-29/30 (20°20'06"S, 43°56'19"W), 1♂ 1imm., 3-20/XI/2007, R. Andrade et al. (IBSP 97952); 1♀, 03-06/X/2011, J. Mascarenhas (IBSP 191320); 1♂, 29/III-03/IV/2012, J. Mascarenhas (IBSP 191321).

#### Distribution.

Known only from the state of Minas Gerais, southeastern Brazil (Fig. 18A–B).

#### Natural history.

All 79 specimens (7♂, 24♀, 48 immature) of *Tisentnops mineiro* sp. n. were collected in 33 caves distributed in rock outcrops in rupestrian fields found in mountain peaks of the Atlantic Forest and lowland areas of regenerated forest or grasslands. Unlike *Tisentnops onix* sp. n. from limestone caves, *Tisentnops mineiro* sp. n. was only found in iron caves. Additionally, *Tisentnops mineiro* sp. n. was found in palaeoburrows “*Paleotoca*” (natural shelter of extinct mammals, e.g. Giant Armadillos, see Bittencourt et al. 2015, figs 5–6) in Cave AP-38 in the municipality of Santa Bárbara. *Tisentnops mineiro* sp. n. were found on the ground, under rocks in aphotic zones with high relative humidity (≥ 98%). *Tisentnops mineiro* sp. n. is a troglobite spider restricted to caves from target mining areas and regions of iron formations with high economic interest.

### Tisentnops onix sp. n.

Taxon classificationAnimaliaAraneaeCaponiidae

http://zoobank.org/1EDE3574-70B1-43DC-8A92-0316D2C8A4CD

Figures 4F–J , 5 , 17B–D

#### Types.

Male holotype from Maciço da Gruta da Taboa, Cave 64 (19°28'35"S, 44°55'34"W), Sete Lagoas, Minas Gerais, Brazil, 26.II.2015, F. Bondezan col., deposited in IBSP 186339; female paratype from Gruta da Taboa (19°28'01"S, 44°19'0"W), Fazenda Taboa, Sete Lagoas, Minas Gerais, 31.IV.2014, L.S. Carvalho col., deposited in UFMG 15527.

#### Etymology.

The specific name refers to the shape of the black spot on the ocular area which resembles a lozenge-shaped onyx jewel.

#### Diagnosis.

*Tisentnops onyx* sp. n. differs from *Tisentnops mineiro* sp. n. by the presence of two eyes (Fig. 5E) and by a longer and more slender internal V-shaped dorsal fold in the female genitalia (Fig. 4F–G).

#### Description.

**Male (holotype).** Total length 3.7. Carapace 1.8 long, 1.4 wide. Coloration as in *Tisentnops mineiro* sp. n., except cephalothorax red-brown, with black spot in the ocular area, and legs olive-brown. Leg measurements: I: femur 1.2/ patella 0.6/ tibia 1.0/ metatarsus 0.5/ tarsus 0.5/ total 3.8; II: 1.05/ 0.55/ 0.6/ 0.45/ 0.45/ 3.1; III: 0.9/ 0.4/ 0.6/ 0.5/ 0.5/ 2.9; IV: 1.2/ 0.6/ 1.2/ 0.6/ 0.7/ 4.3. Legs with long trichobothria. Elongate raised sockets as in *Tisentnops mineiro* sp. n. Palpal cymbium twice the length of the bulb, bulb enlarged distally, next to the base of the embolus. Embolus curved, half the length of bulb and narrow medially (Fig. 4F–H).

**Female (paratype)**. Total length 4.7. Carapace 1.9 long, 1.4 wide. Coloration as in male, except sternum, endites and chelicerae red-brown (Fig. 5A–E). Leg measurements: I: femur 1.3/ patella 0.6/ tibia 1.0/ metatarsus 0.5/ tarsus 0.5/ total 3.9; II: 1.1/ 0.5/ 0.8/ 0.5/ 0.4/ 3.3; III: 0.9/ 0.5/ 0.6/ 0.6/ 0.4/ 3.0; IV: 1.2/ 0.5/ 1.2/ 0.9/ 0.7/ 4.5. Legs with tricobothria as in male (Fig. 5H). Elongated raised sockets as in male. External female genitalia weakly sclerotized, transparent so that receptaculum is visible (Fig. 5F). Internal female genitalia with a straight anterior margin of the hyaline membrane that covers the elongate sclerotized bars, an elongate, membranous anteromedian receptaculum with a narrow base, and a slender V-shaped dorsal fold (Fig. 4F–G).

#### Other material examined.

None.

#### Distribution.

Known only from the type locality in the state of Minas Gerais, southeastern Brazil (Fig. 18A).

#### Natural history.

*Tisentnops onix* sp. n. is a troglobite spider. The female was collected from a cave, 15–20 meters from the entrance, under a rock in the aphotic area. The soil is very humid, composed of large and small rocks (Fig. 17A), is not sandy and lacks bat guano. Four people were collecting for at least 3 hours in this cave, and this was the only Caponiidae collected. Other arachnids were also captured: two species of spiders from the Ctenidae, *Enoploctenus* Simon and of *Ctenus* Keyserling, many specimens Mesabolivar aff. togatus (Pholcidae), and specimens of *Loxosceles similis* Moenkhaus (Sicariidae). Additionally, one species of *Charinus* Simon (Amblypygi) and Pseudoscorpiones of the family Cheliferidae were captured. No natural history data was obtained from the male specimen, but it was found in the aphotic area of Cave 64.

### Taintnops

Taxon classificationAnimaliaAraneaeCaponiidae

Platnick

Taintnops Platnick, 1994b: 9 (type species by original designation Taintnops goloboffi Platnick).

#### Diagnosis.

**Figure 6.**
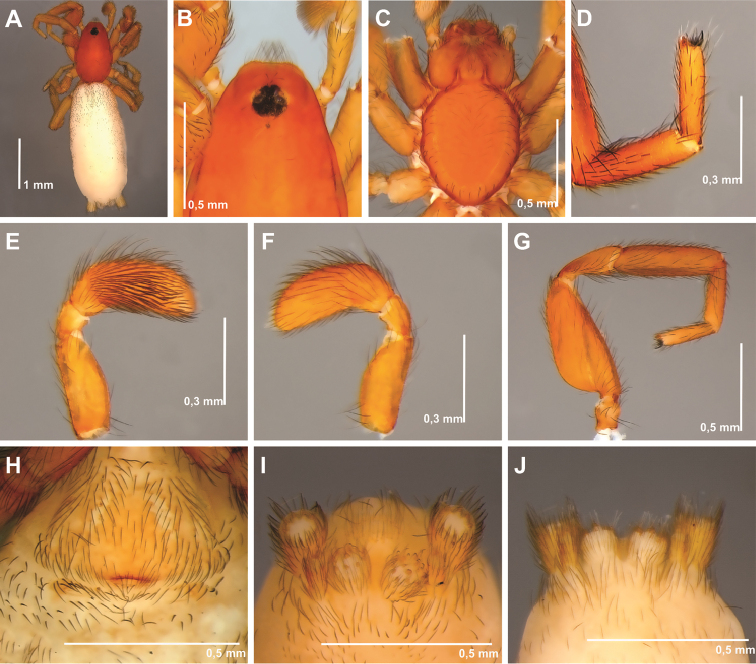
*Taintnops paposo* sp. n. from Taltal, Antofagasta, Chile, female paratype (**A–J**) **A** habitus, dorsal view **B** carapace, ocular area, dorsal view **C** carapace, ventral view **D** leg I, metatarsus and tarsus distal, retrolateral view **E** pedipalp, prolateral view **F** same, retrolateral view **G** leg I, prolateral view **H** genital area, ventral view **I** spinnerets, posterior view **J** same, ventral view.

**Figure 7.**
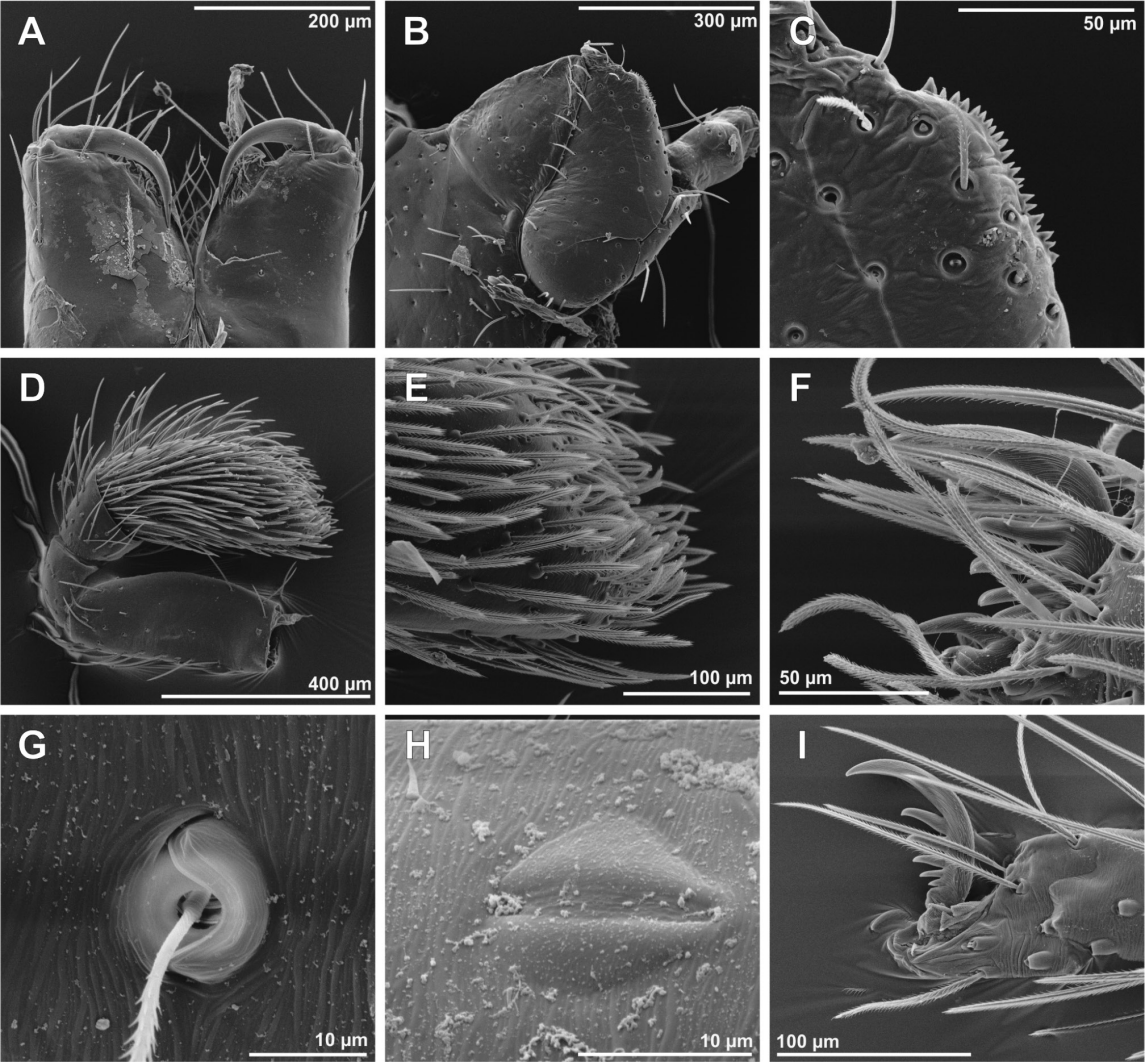
SEM images of *Taintnops paposo* sp. n., female paratype (**A–I**) **A** chelicerae, ventral view **B** left endite and labium, ventral view **C** left serrula, ventral view **D** left pedipalp, prolateral view **E** same, distal area, prolateral view **F** paired claws on right leg I, prolateral view **G** trichobotria base on leg II, dorsal view **H** sensilla on leg II, dorsal view **I** paired claws on right leg IV, prolateral view.

Members of *Taintnops* can be separated from other caponiids as follows: from Nopinae genera by having entire, rather than subsegmented tarsi, from *Caponia*, *Calponia*, *Carajas* gen n., *Nasutonops* gen. n. and *Notnops* by having only two eyes (Fig. 6B), from *Diploglena* and *Tisentnops* by the normal (rather than anteriorly expanded) palpal endites (Figs 6C, 7B), and from *Caponina* by the pear-shaped bulb and the short, distally situated embolus of males (Platnick 1994b, figs 22–24) and the distinct pad of shortened setae on the distodorsal surface of the female palpal tarsus (Figs 6E, 7D–E).

#### Description.

Described by Platnick (1994b), but new details from SEM data and fresh specimens are presented here. Two eyes surrounded by a black area (Fig. 6B). Clypeus with at least six pairs of long setae (Figs 6B–C). Chelicerae (Fig. 7A) with short tooth-shaped tip on median lamina and white membranous lobe opposite the tip of the cheliceral fang. Endites acuminate, converging, not touching and not protuberant posteriorly (Figs 6C, 7B), serrula distal, with a single tooth row (Fig. 7C). Labium covered with plumose setae (Fig. 7B). Sternum oval, cuticle with long black setae (Fig. 6C). Female palp with dense patch of setae prolaterally and distinct distodorsal pad of shortened setae (Figs 6E–F, 7D–E). Legs pilose (Figs 6D, G), paired claws I-II with six teeth, and unpaired claw short without teeth (Fig. 7F), paired claws III-IV with five teeth (Fig. 7I), and unpaired claws short. Tarsal organ not found. Tibiae with row of five dorsal and two prolateral trichobothria; metatarsi and tarsi with two prolateral and two dorsal pairs of trichobothria, with a semicircular rim bearing few ridges (Fig. 7G), elongate and slightly sulcate sensilla (Fig. 7H). Six spinnerets in typical caponiid arrangement; anterior laterals greatly reduced and the same length as posterior laterals, females apparently with one major ampullate gland spigot and two smaller piriform gland spigots; posterior medians with single, enlarged, medially situated spigot presumed to serve the minor ampullate gland and 10–12 smaller, more peripheral spigots, arranged in a ring, presumed to serve the aciniform glands; posterior laterals with peripheral ring of 8–10 presumed aciniform gland spigots (Fig. 6I–J). External female genitalia with weakly sclerotized anterior plate. Internal female genitalia consisting of large, oval anteromedian membranous receptaculum, and V-shaped posterior bar with wide ends (Fig. 16E; Platnick 1994b: fig. 25).

### Taintnops paposo sp. n.

Taxon classificationAnimaliaAraneaeCaponiidae

http://zoobank.org/A882F6D3-9571-41F0-855E-30D498B58262

Figures 6 , 7 , 16E

#### Types.

Holotype and paratype females from Reserva Nacional Paposo (24°57,82'S, 70°27,961'W), 52m asl, Taltal, Antofagasta Province, Chile, 16/VII/2012, A.D. Brescovit, A.J. Santos & A. Taucare-Rios col., deposited in IBSP 166983 and 166984, respectively.

#### Etymology.

The specific name is a noun in apposition taken from the type locality.

#### Diagnosis.

Females differ from those of *Taintnops goloboffi* Platnick (see Platnick 1994b: fig. 25) by the oval anteromedian receptaculum lacking posterior extensions (Fig. 16E)

#### Description.

**Male.** Unknown.

**Female (holotype).** Total length 3.9. Carapace 1.3 long, 0.8 wide. Carapace, mouthparts and sternum reddish. Border of eyes black. Legs and pedipalp orange. Abdomen uniformly creamy white, with epyginal area orange (Fig. 6H). Leg measurements: I: femur 0.85/ patella 0.4/ tibia 0.55/ metatarsus 0.45/ tarsus 0.3/ total 2.55; II: 0.75/ 0.4/ 0.55/ 0.45/ 0.25/ 2.40; III: 0.6/ 0.3/ 0.4/ 0.35/ 0.3/ 1.95; IV: 0.9/ 0.4/ 0.7/ 0.6/ 0.35/ 2.45. External female genitalia with weakly sclerotized anterior plate, and posterior border of genital opening strongly sclerotized (Fig. 6H). Internal genitalia with oval and elongate membranous anteromedian receptaculum not extending posteriorly, lacking sclerotized posterior extensions (Fig. 16E).

#### Other material examined.

None.

#### Distribution.

Known only from Taltal area, in the Antofagasta Region.

#### Natural history.

Both specimens were collected under rocks during the day. Silk retreats were not observed in the area.

### Nasutonops gen. n.

Taxon classificationAnimaliaAraneaeCaponiidae

http://zoobank.org/DB6219F6-3347-4AFC-A52B-278E32FA652D

#### Type species.

*Nasutonops xaxado* sp. n.

#### Etymology.

The generic name comes from the Latin words *nasutus* (with a large nose) combined with *nops* (less eyes), and is masculine in gender.

#### Diagnosis.

**Figure 8.**
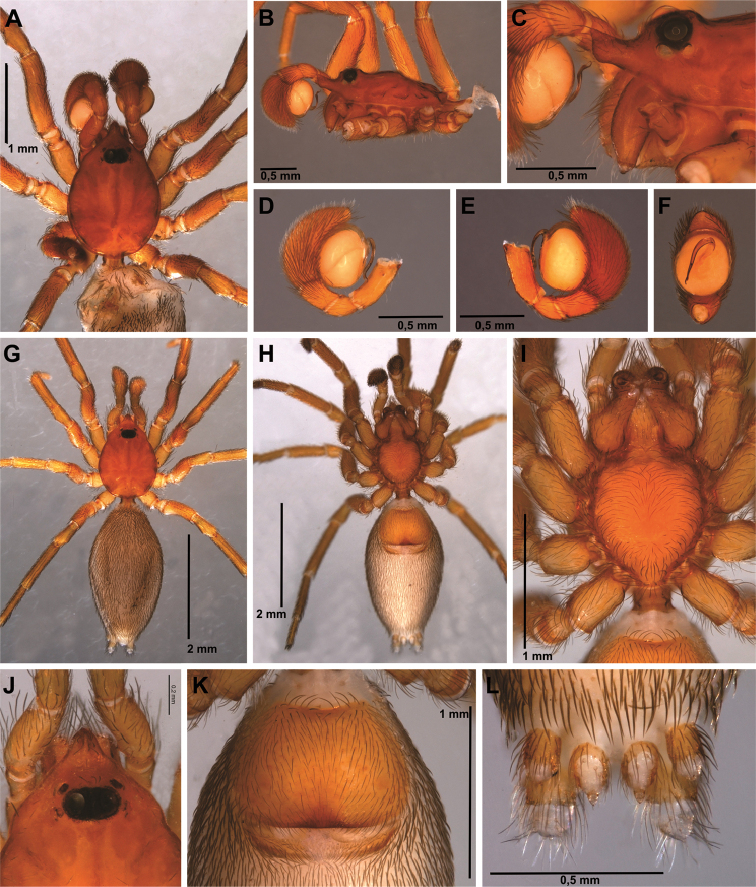
*Nasutonops xaxado* sp. n., male from Serra Talhada, Pernambuco (**A–F**) female, same locality (**G–L**) **A** habitus, dorsal view **B** carapace, lateral view **C** ocular area, lateral view **D** left pedipalp, prolateral view **E** same, retrolateral view **F** same, ventral view **G** habitus, dorsal view **H** same, ventral view **I** carapace, ventral view **J** ocular area, dorsal view **K** genital area, ventral view **L** spinnerets, ventral view.

Males and females can be easily distinguished from all other known caponiids by the presence of a clypeal horn, projected distally (Fig. 8A, C, J). The genus resembles species of *Caponina* by having the palp with a globose bulb and an elongate and curved embolus in males (see Platnick 1994, figs 26–28) and by having a similar pair of boomerang-shaped sclerotized bars in the female genitalia (see Platnick 1994, figs 22–25); however, it differs by the flattened base of the embolus originating from the distal area of the male bulb (Fig. 11D–E), rather than the median area as in *Caponina*. The female differs by the strongly sclerotized transverse internal fold of the female genitalia, which is observed by transparecy on the posterior border of the anterior plate (Figs 8K, 11A–B, F–I; 12H–J), which is not apparent in any *Caponina* species. These three structures support the monophyly of *Nasutonops*.

#### Description.

**Figure 9.**
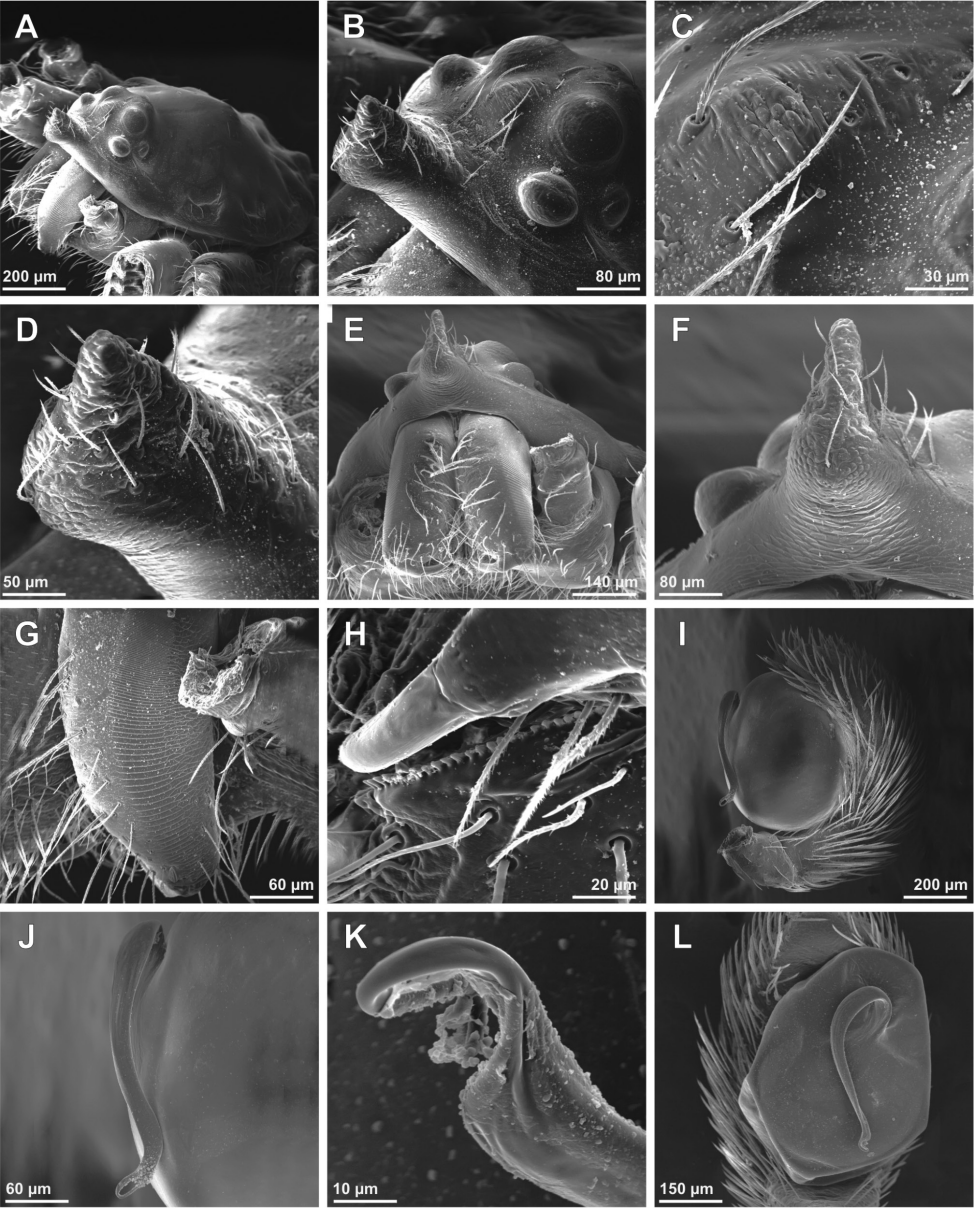
SEM images of *Nasutonops xaxado* sp. n., male from Serra Talhada, Pernambuco (**A–L**) **A** carapace, lateral oblique view **B** ocular area, lateral oblique view **C** striated area between posterior eyes, detail, dorsal view **D** clypeal projection, lateral view **E** same with chelicerae, anterior view **F** same, anterior view **G** chelicerae, stridulatory area, lateral view **H** serrula, lateral view **I** left pedipalp, retrolateral view **J** embolus, retrolateral view **K** tip of embolus, distal area **L** bulb, ventral view.

**Figure 10.**
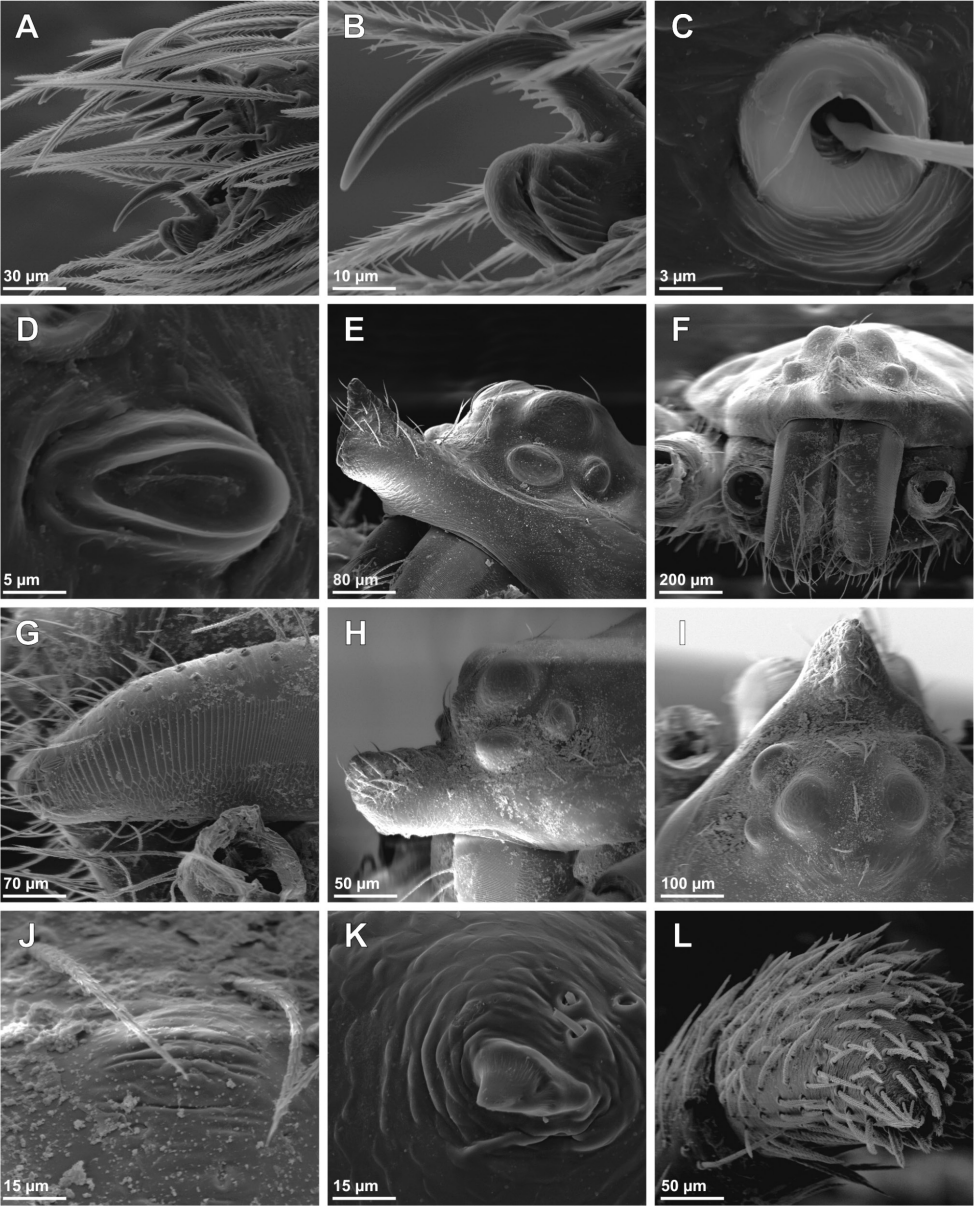
SEM images of *Nasutonops xaxado* sp. n., male (**A–D**) and female (**E–L**) from Serra Talhada, Pernambuco. **A** paired claws on right leg II, prolateral view **B** unpaired claw on right leg II, prolateral view **C** trichobothria base on right leg II, dorsal view **D** tarsal organ on right leg II, dorsal view **E** ocular area, lateral oblique view **F** same, anterior view **G** chelicerae, stridulatory area, lateral view **H** clypeal projection, lateral view **I** same, dorsal view **J** striated area between posterior eyes, detail, dorsal view **K** stridulatory pick on left pedipalp, prolateral view **L** distal area of left pedipalp, anterior view.

**Figure 11.**
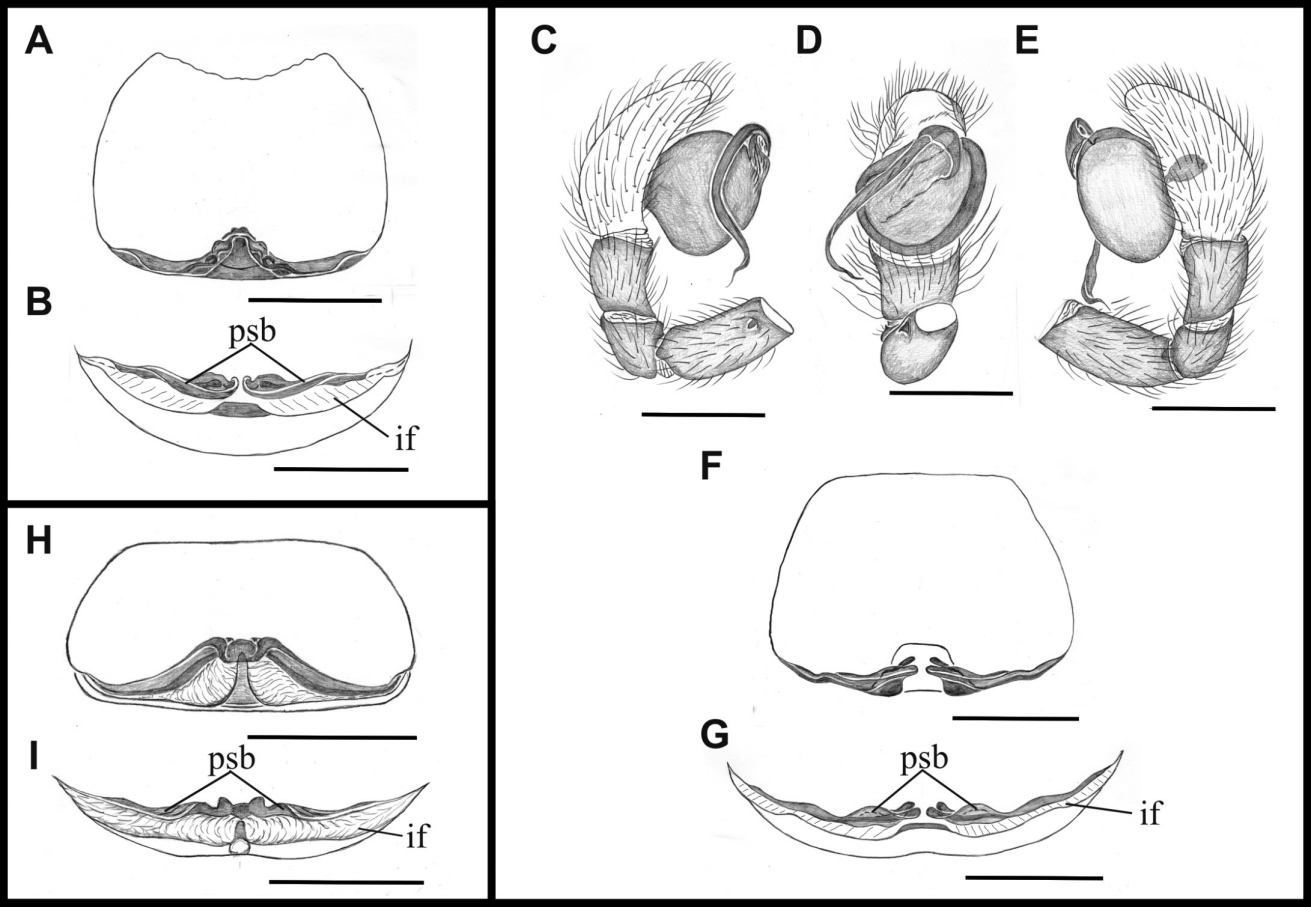
*Nasutonops xaxado* sp. n., female from Serra Talhada, Pernambuco (**A–B**) *Nasutonops chapeu* sp. n., male and female from Irecê, Bahia (**C–G**), *Nasutonops sincora* sp. n., female from Contendas do Sincorá, Bahia (**H–I**) (**A–B, F–G, H–I**) female internal genitalia **A, F, H** dorsal view **B, G, I** anterior view (**C–E**) male palp, A prolateral view **B** ventral view **D** prolateral view (if = internal transversal fold, psb = pair of sclerotized bars). Scale bars: **A–G** 0.5 mm.

**Figure 12.**
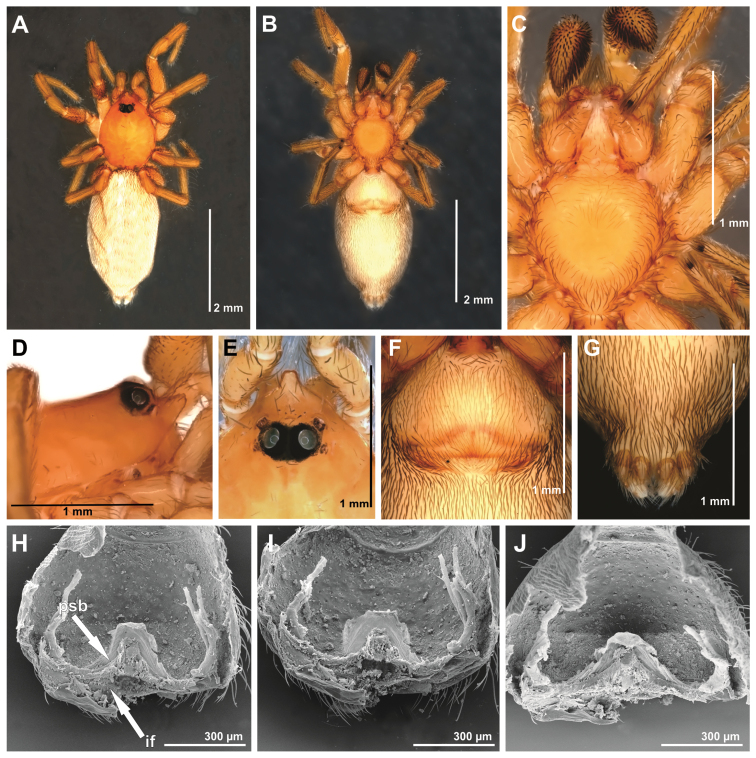
*Nasutonops sincora* sp. n., female from Contendas do Sincorá, Bahia (**A–G**). **A** habitus, dorsal view **B** same, ventral view **C** carapace, ventral view **D** ocular area, lateral view **E** same, dorsal view **F** genital area, ventral view **G** spinnerets, ventral view. *Nasutonops xaxado* sp. n. (**H–J**) **H** SEM images of internal genitalia, dorsal view **I** same, posterior view **J** same, anterior view (if = internal transversal fold; psb = pair of sclerotized bars). Scale bars: **A–G** 0.5 mm.

Moderate-sized caponiids with six eyes (Fig. 8A, G–H, J). Carapace oval, anteriorly narrowed to half its maximum width, pars cephalica depressed behind cephalic area, depressed between coxa of endite and coxa I, pars thoracica medially higher than laterally or posteriorly, gradually sloping laterally and posteriorly (Fig. 8B); cuticle smooth; few dorsally directed strong bristles on the clypeal area; carapace smooth; thoracic groove almost absent (Fig. 8A, G). Clypeal horn, distally conic, striated anteriorly and posteriorly, shorter in females (Figs 8C, J; 9A–F; 10E–F, H–J). Six eyes, medians largest and more elevated than laterals (Figs 8C, 9A–B), dark, separated by almost their radius, surrounded by black pigment; laterals white and oval, posteriors half the size of anteriors (Figs 8J, 10I). Cheliceral paturon with long and strong bristles medially (Figs 9E, 10F); base of fang unmodified; median lamina short, with irregular anteromedian tip; most of the space between the lamina and base of fang occupied by white membranous lobe; lateral surface with large stridulatory ridges in males and females (Figs 9G; 10G), pick on prolateral side of palpal femur, triangular, situated at approximately one-fifth of femur length (Figs 10K, 11C–D). Endites convergent, acuminate, not touching, covered with many long basal setae that shorten distally (Fig. 8I), with strong and long distal serrula consisting of a single tooth row with more than 20 teeth (Fig. 9H). Labium triangular, fused to sternum, covered with many scattered setae (Fig. 8H–I); labrum short, narrow, slightly elevated. Sternum longer than wide, smooth, without radial furrows between coxae, covered with scattered long setae, not fused to carapace (Fig. 8I); cephalothoracic membranes without epimeric sclerites, but long triangular sclerites extend from sternum between coxae I and II, II and III, and III and IV, shorter triangles extend to each coxae. Leg formula 4123; legs without spines; metatarsi and tarsi entire, without subsegmentation or membranous processes; tarsi with three claws; paired claws with approximately ten teeth (more on leg I-II), distal teeth largest; unpaired claw shorter than paired ones, with five minuscule teeth (Fig. 10A–B). Tibiae with trichobothria in a double row, metatarsi and tarsi with trichobothria in single row, bases almost smooth, with strong external border (Fig. 10C); tarsal organ exposed, oval, not elevated, consisting of two oval, sclerotized laminae (Fig. 10D); female palpal tarsus elongate, prolateral surface densely covered with setae, without claw (Figs 10L; 12C). Abdomen immaculate and pilose; epigastric plate sclerotized (Figs 8G–H; 12A–B), two pairs of small respiratory spiracles. Six spinnerets (Figs 8L, 12G) in typical caponiid arrangement, anterior laterals shorter than posterior laterals, with one major ampullate gland spigot and at least three smaller piriform gland spigots; posterior medians with single, very thick, medially situated spigot presumed to serve minor ampullate gland, and 5-6 smaller, more peripheral spigots arranged in a ring, presumed to serve aciniform glands; posterior laterals with 8-10 central and peripheral aciniform gland spigots. Male palpal femur twice the length of the tibia, patella shorter than the tibia, unmodified; cymbium ovoid, prolateral surface densely covered with strong setae; bulb globose; embolus long, curved at base, flattened, with small teeth in the distal third, narrowed distally, with sinuous tip (Figs 8B–F; 9I–L). External female genitalia with large, rectangular, sclerotized anterior plate, and postepigastric scutum represented by a pair of narrowed sclerites, represented by a transparent area on an internal strongly sclerotized trasverse fold on the posterior border of the anterior plate (Figs 8K; 12 F). Internal female genitalia consist of a pair of boomerang-shaped sclerotized bars, that may or may not converge anteriorly, associated with the uterus externus short but with a wide base occupying almost the entire width of the sclerotized bars; strongly sclerotized transverse fold runs along nearly all of the epigastric area, reaching the posterior ends of the sclerotized bars (Figs 11A–B, F–I; 12H–J).

#### Distribution.

Known only from Brazilian Caatinga in the states of Bahia and Pernambuco, Brazil.

### Nasutonops xaxado sp. n.

Taxon classificationAnimaliaAraneaeCaponiidae

http://zoobank.org/C40D5196-C43E-4B93-8EF6-7CA9F0BAA242

Figures 8 , 9 , 10 , 11A–B , 12H–J

#### Types.

Male holotype and female paratype from Serra Talhada (7°59'9"S, 38°17'45"W), Pernambuco, Brazil, 2008-2009, H. Amorin col., deposited in IBSP 166987 and 166988, respectively.

#### Etymology.

The specific name is a nickname of the type locality, known as the ‘’Capital do Xaxado’’. The Xaxado is a popular dance in this region.

#### Diagnosis.

Females of *Nasutonops xaxado* sp. n. resemble those of *Nasutonops chapeu* sp. n. in lacking the connection of a pair of sclerotized bars in the female internal genitalia (Fig. 11F–G), but can be distinguished by the enlarged anterior ends of the pair of elongate sclerotized bars and the short posterior ends (Fig. 11A–B). Males differ by their shorter embolus and curved tip (Figs 8F, 9J–K).

**Male (Holotype).** Total length 3.80, with clypeal horn. Carapace 1.7 long, 1.2 wide. Coloration: carapace orange-reddish, except apex of endites and labium white, legs and palps orange. Eye median diameters 0.3, interdistances 0.15. Leg measurements: I: femur .1.1/ patella 0.5/ tibia 0.8/ metatarsus 0.7/ tarsus 0.4/ total 3.5; II: 1.1/ 0.5/ 0.7/ 0.7/ 0.4/ 3.4; III: 0.9/ 0.5/ 0.6/ 0.6/ 0.5/ 3.1; IV: 1.1/ 0.6/ 1.0/ 0.9/ 0.6/ 4.2. Abdomen gray with epiandric plate and spinnerets orange. Palpal cymbium not covered with thick layer of distal setae (Fig. 9I).

**Female (allotype).** Total length 4.5, with clypeal horn. Carapace length 1.7 width 1.2. Coloration as in male. Eye median diameters 0.3, interdistances 0.2. Leg measurements: I: femur .1.0/ patella 0.5/ tibia 0.8/ metatarsus 0.6/ tarsus 0.5/ total 3.4; II: 1.0/ 0.5/ 0.7/ 0.7/ 0.5/ 3.4; III: 0.9/ 0.4/ 0.6/ 0.6/ 0.4/ 2.9; IV: 1.0/ 0.5/ 0.8/ 0.9/ 0.5/ 3.7. Internal genitalia with enlarged anterior ends and short posterior ends of the pair of sclerotized bars (Figs 11A–B, 12H–J).

#### Note.

Left metatarsus and tarsus IV of male absent.

#### Other material examined.

BRAZIL: **Pernambuco**, Serra Talhada (7°59'9"S, 38°17'45"W), 1♂ 1♀, 2008-2009, H. Amorin col. (IBSP 166989; 166990, respectively, both partially used in SEM).

#### Distribution.

Known only from the type locality in the Brazilian Caatinga.

#### Natural history.

The specimens were collected in soil with pitfall traps.

### Nasutonops chapeu sp. n.

Taxon classificationAnimaliaAraneaeCaponiidae

http://zoobank.org/CDDB6E7F-32DE-488A-9E4A-6F9E83D74711

Figure 11C–G

#### Types.

Male holotype from Parque Estadual Morro do Chapéu (11°29'19,2"S, 41°15'27,6"W), Morro do Chapéu, Bahia, 21.I.2012, 1097 m asl., I.L.F. Magalhaes et al. col, and female allotype from Área da Mineradora Galvani, Irecê (11°18'14"S, 41°51'21"W), Bahia, 27.VI.-27.IX.2011, M.C. Nunes col., deposited in IBSP 161985 and 161986, respectively.

#### Note.

Although the male and female were not collected from the exact same locality, both specimens were collected near one another in the state of Bahia, and they have the same body coloration.

#### Etymology.

The specific name is an apposition to the type locality.

#### Diagnosis.

Females of *Nasutonops chapeu* sp. n. resemble those of *Nasutonops xaxado* sp. n. in lacking the connection of the pair of elongate sclerotized bars in the female internal genitalia (Fig. 11F–G), but can be distinguished by the narrow anterior ends and slender posterior ends of the pair of sclerotized bars (Fig. 11F–G). Males differ by the elongate embolus with a slender and sinuous tip (Fig. 11C–E).

#### Description.

**Male (holotype)**. Total length 3.2, with clypeal horn. Carapace 1.3 long, 1.05 wide. Coloration of body as in *Nasutonops xaxado* sp. n., except abdomen grayish. Eye median diameters 1.0, inter distances 0.6. Leg measurements: I: femur 1.0/ patella 0.4/ tibia 0.75/ metatarsus 0.55/ tarsus 0.3/ total 3.0; II: 0.9/ 0.4/ 0.65/ 0.55/ 0.3/ 2.8; III: 0.75/ 0.35/ 0.5/ 0.5/ 0.25/ 2.35; IV: 0.95/ 0.4/ 0.8/ 0.7/ 0.45/ 3.3.

**Female (allotype)**. Total length 4.3, with clypeal horn. Carapace 1.55 long, 1.1 wide. Coloration as in male. Eye median diameters 1.0, inter distances 0.8. Leg measurements: I: femur 1.1/ patella 0.45/ tibia 0.75/ metatarsus 0.65/ tarsus 0.4/ total 3.35; II: 1.0/ 0.4/ 0.75/ 0.6/ 0.45/ 3.2; III: 0.8/ 0.35/ 0.6/ 0.6/ 0.35/ 2.7; IV: 1.1/ 0.55/ 0.9/ 0.9/ 0.5/ 3.95. Internal genitalia with long pair of elongate sclerotized bars with narrow anterior ends and slender posterior ends, and with an enlarged area on the anterior third (Fig. 11G).

#### Other material examined.

None.

#### Distribution.

Known only from the type locality in the Brazilian Caatinga from the state of Bahia.

### Nasutonops sincora sp. n.

Taxon classificationAnimaliaAraneaeCaponiidae

http://zoobank.org/22845E0E-E92D-4F8C-B8D9-36CDD2FC3BC2

Figures 11H–I , 12A–G

#### Types.

Female holotype from Floresta Nacional Contendas do Sincorá (13°46'–14°00'S, 41°03'–41°10'W), Contendas do Sincorá, Bahia, Brazil, X.2007–X.2008, Y.G. Santos col., deposited in IBSP 126918.

#### Etymology.

The specific name is an apposition to the type locality.

#### Diagnosis.

The female of *Nasutonops sincora* sp. n. differs from others females of the genus by the elevated area of the pair of transverse elongate sclerotized bars that are connected anteriorly, forming a rounded, small plate (11H–I) in the female genitalia.

#### Description.

**Male.** Unknown.

**Female (holotype).** Total length 4.5, with shortest clypeal horn. Carapace 1.7 long, 1.2 wide. Coloration: cephalothorax and legs orange, ocular area black, abdomen cream, covered with gray hairs, genital area orange and spinnerets yellow (12 A–B). Clypeal horn truncate at tip (Fig. 12D–E). Eye median diameters 0.12, interdistances 0.8. Leg measurements: I: femur 1.1/ patella 0.55/ tibia 0.8/ metatarsus 0.65/ tarsus 0.45/ total 3.55; II: 1.05/ 0.5/ 0.8/ 0.6/ 0.4/ 3.35; III: 0.75/ 0.4/ 0.55/ 0.65/ 0.4/ 2.75; IV: 1.25/ 0.55/ 1/ 0.8/ 0.5/ 4.1. External genitalia with receptacula visible through transparent area (Fig. 12F). Internal genitalia with a pair of transverse elongate sclerotized bars, connected anteriorly, forming a rounded small plate; internal fold between the pair of sclerotized bars (Fig. 11I).

#### Other material examined.

None.

#### Distribution.

Known only from the type locality in the Brazilian Caatinga from the state of Bahia.

### Carajas gen. n.

Taxon classificationAnimaliaAraneaeCaponiidae

http://zoobank.org/90E1A654-6D56-4276-97A7-6C85489B7975

#### Type species.

*Carajas paraua* sp. n.

#### Etymology.

The generic name is an apposition to the type locality where all known specimens were collected and is masculine in gender.

#### Diagnosis.

**Figure 13.**
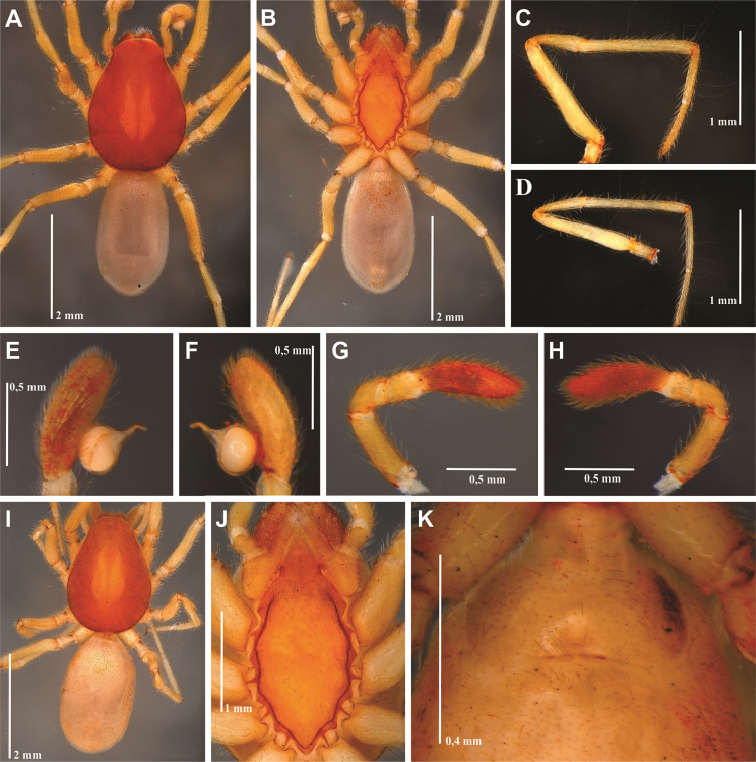
*Carajas paraua* sp. n., male (**A–F**) and female (**G–K**) from Parauapebas, Pará. **A** habitus dorsal view **B** same, ventral view **C** left leg I, prolateral view **D** left leg IV, prolateral view **E** left pedipalp, prolateral view **F** same, retrolateral view **G** left pedipalp prolateral view **H** same, retrolateral view **I** habitus, dorsal view **J** carapace, ventral view **K** genital area, ventral view.

**Figure 14.**
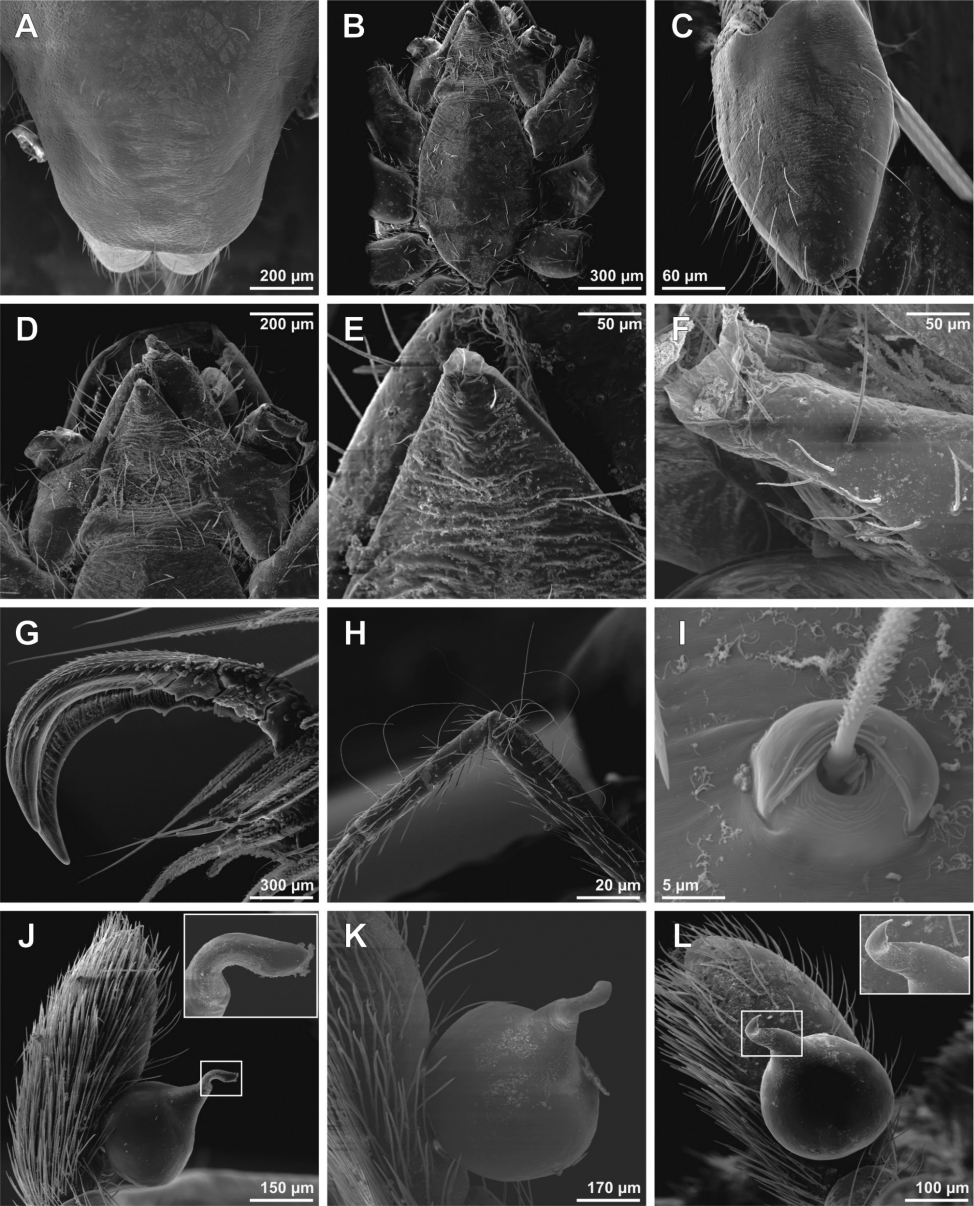
SEM images of *Carajas paraua* sp. n., male from type locality. **A** carapace, ocular area, dorsal view **B** carapace, ventral view **C** left chelicerae, prolateral view **D** mouthparts, ventral view **E** labium, distal area, ventral view **F** labium, distal area, prolateral view **G** paired claws on right leg IV, prolateral view **H** right tibiae and metatarsus I showing long trichobotria, prolateral view **I** tricobothria base on right leg I, dorsal view **J** pedipalp, prolateral view (inset: detail of embolus) **K** bulb, dorsal view **L** same,ventral view (inset: detail of embolus).

**Figure 15.**
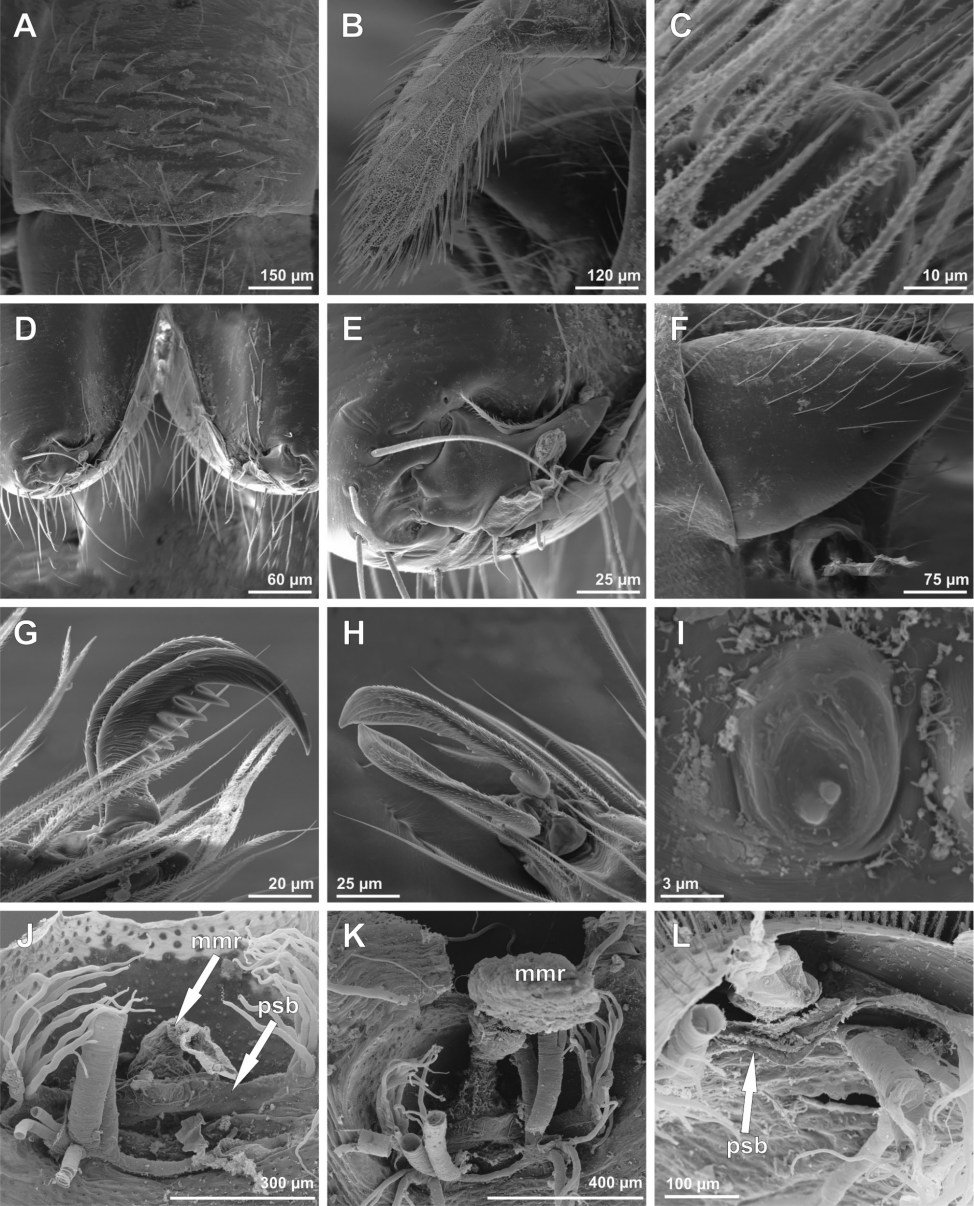
SEM images of *Carajas paraua* sp. n., three females (IBSP 161403) specimen 1 **(A–J)**, specimen 2 **(K)**, specimen 3 **(L) A** carapace, ocular area, dorsal view **B** pedipalp, retrolateral view **C** same, distal area, prolateral view **D** chelicerae, ventral view **E** same, distal area showing fang, ventral view **F** same, prolateral view **G** claws on left leg I, prolateral view **H** claws on left leg IV, dorsal view **I** tarsal organ on left leg IV, dorsal view **J** internal genitalia, dorsal view **K** same, dorsal view **L** same, anterior view (if = internal transversal fold; mmr = membranous anteromedian receptaculum; psb = pair of sclerotized bars).

Members of *Carajas* gen. n. can be easily separated from other caponiid genera by having anteriorly and posteriorly strongly projected endites (Figs 13J, 14D), a very short cheliceral fang (Fig. 15D–E), absence of unpaired claws on all legs, and the posterior paired claws with distal tip thickened and covered with dense and short bristles (Fig. 14G, 15H).

#### Description.

**Figure 16.**
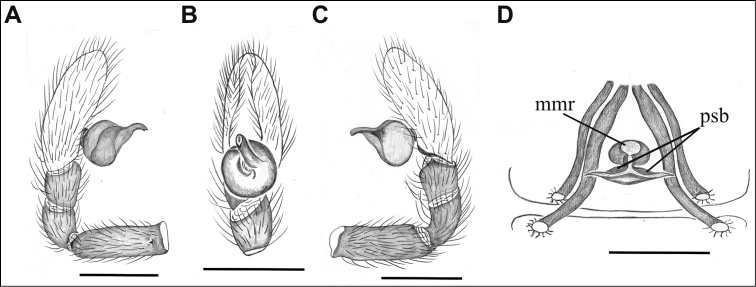
*Carajas paraua* sp. n., male from IBSP 161191 (**A–C**) and female from IBSP 191285 (**D**). **A** left pedipalp, prolateral view **B** same, ventral view **C** same, retrolateral view **D** internal genitalia and tracheae tubes, dorsal view (mmr = membranous anteromedian receptaculum; psb = pair of sclerotized bars). Scale bars: **A–D** 0.5 mm.

Moderate-sized caponiids (Fig. 13A, I). Carapace oval, anteriorly narrowed to less than half of its maximum width, pars cephalica rounded, pars thoracica flat medially and sloping posteriorly; cuticle of carapace smooth, covered with short and uniform setae; clypeus almost smooth; thoracic groove inconspicuous, almost obsolete (Fig. 13A, I) . Eyes absent (Figs 13A, I; 14A, 15A). Cheliceral paturon with long, weak bristles, mainly on frontal area; base of fang unmodified; fang short with large opening, median lamina short, occupying a small space by the white membranous lobe; lateral surface with small sulci of stridulatory ridges (Fig. 14C) , pick small on prolateral basal side of palpal femur (Fig. 16A). Endites convergent, strongly projected, extending anteriorly far beyond the anterior margin of the labium, and posteriorly widened, extending far beyond the posterior margin of the labium (Figs 13J, 14D), covered with many small and long setae (Fig. 14D–E), with strong distal serrula consisting of a single tooth row. Labium triangular, strongly fused to sternum, not invaginated at base, covered with a few scattered setae, distal area hyaline (Figs 13B, 14E); labrum elongate, narrow, subtriangular, distally slightly elevated. Sternum longer that wide, without radial furrows between coxae, covered with small and uniform setae, not fused to carapace, with sclerotized lateral and posterior borders (Figs 13B, J; 14B); cephalothoracic membranes without epimeric sclerites, but short triangular sclerites extend from the sternum between coxae of endites and coxa I, I and II, II and III, and III and IV, shorter triangles extend to each coxae, straighter on coxae III and IV, posterior border triangular between coxae IV (Fig. 13B, J). Legs formula 4213, without spines, metatarsi and tarsi entire, without subsegmentation or membranous processes, tarsi with two claws, lack unpaired claws, surrounded with elongate hairs; paired claws I-II with approximately six teeth, distal tip of claw elongate (Fig. 15G), III-IV weakly curved, with 3-4 short and well-separated teeth, distal tip of claw thickened, covered with dense and short bristles (Figs 14G, 15H). Tibiae, metatarsi, and tarsi with trichobothria in a single row, bothrium ridged (Fig. 14I), and very long bristles, several of them longer than metatrsus (Figs 13C–D, 14H); tarsal organ oval, exposed, with concentric ridges and two distal receptors (Fig. 15I); female palpal tarsus three times longer than the tibia, prolateral surface densely covered with setae, without claw (Figs 13G–H, 15B–C). Abdomen without scutum; covered uniformly with short setae, not striated (Fig. 13A–B, H–I). Epigastric region slightly protruding, with two pairs of respiratory spiracles, connected to large tracheal trunks directed anteriorly (Fig. 16D), posterior spiracles connected by rebordered groove extending parallel with the anterior spiracles; postepigastric scutum not fused to epigastric scutum. Males and females with six spinnerets in typical caponiid arrangement, anterior laterals with single, presumably major ampullate gland spigot, posterior medians with large, flattened minor ampullate gland spigot and posterior laterals with approximately ten aciniform gland spigots. Male palpal patella and tibia shorter that femur, unmodified; cymbium ovoid, elongate, prolateral surface densely covered with strong setae; bulb globose; embolus short, ribbon-like at base, slightly curved distally, tip enlarged and rounded (Figs 13E–F, 14J–L, 16A–C). External female genitalia with postepigastric scutum represented only by a basal sclerotized band and part of receptaculum visible through transparent area (Fig. 13K). Internal female genitalia with a membranous anteromedian receptaculum formed by a short unsclerotized median duct with a wide base leading to a large, globose sac. This sac is associated with elongate, sclerotized transverse bars with a dorsally projected anterior margin (Figs 15J–L, 16D).

#### Distribution.

Known only from caves in Carajás region, in the state of Pará, Brazil.

### Carajas paraua sp. n.

Taxon classificationAnimaliaAraneaeCaponiidae

http://zoobank.org/DC7B7823-37B7-47A8-AA70-1D5BD4749DC1

**Figure 17.**
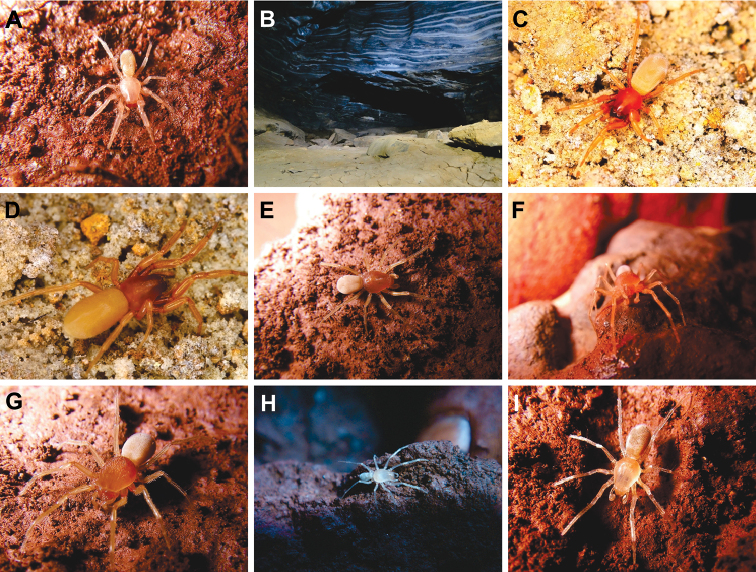
*Tisentnops mineiro* sp. n. (**A**), *Tisentnops onix* sp. n. (**B–D**), *Carajas paraua* sp. n. (**E–I**). **A** adult female on the soil **B** Gruta da Taboa, Minas Gerais, Brazil, showing the rocks on the soil where specimens of *Tisentnops onix* sp. n. were collected **C** adult female on the soil **D** same **E** adult female on rock **F** adult male on rock **G** adult female on rock **H** immature on rock **I** same.

Figures 13 , 14 , 15 , 16A–D , 17E–I

#### Types.

Male holotype and female allotype from Gruta N5S8 (06°06'29"S, 50°07'57"W), Flona de Carajás, Parauapebas, Pará, Brazil, 7-12.X.2008, R. Andrade, deposited in IBSP 191287.

#### Etymology.

The specific name is a noun in apposition taken from the Brazilian Tupi Indian language that means parrot (‘’Papagaio’’ in Portuguese) and refers to this common bird in the region of Parauapebas.

#### Diagnosis.

With the characters of the genus and genitalia as above described.

#### Description.

**Male (holotype).** Total length 4.2. Carapace 2.1 long, 1.5 wide. Coloration: cephalothorax uniformly orange-reddish, except border of sternum brown. Legs and palps yellow. Abdomen uniformly grayish. Leg measurements: I: femur 1.8/ patella 1.0/ tibia 1.4/ metatarsus 1.1/ tarsus 1.0/ total 6.3; II: 1.6/ 1.0/ 1.4/ 1.0/ 0.9/ 5.9; III: 1.5/ 0.7/ 1.1/ 1.0/ 0.8/ 4.1; IV: 2.0/ 1.0/ 2.1/ 1.3/ 1.0/ 7.4. Palp as in figures 13D–E, 14J–L and 16A–C.

**Female (allotype).** Total length 4.3. Carapace 2.1 long, 1.5 wide. Coloration as in male. Leg measurements: I: femur .1.3/ patella 0.7/ tibia 1.1/ metatarsus 0.7/ tarsus 0.6/ total 4.4; II: 1.1/ 0.6/ 0.9/ 0.7/ 0.6/ 3.9; III: 1.0/ 0.6/ 0.8/ 0.7/ 0.5/ 3.6; IV: 1.4/ 0.8/ 1.4/ 0.9/ 0.7/ 5.2. Internal genitalia as described for the genus (Fig. 16D).

#### Other material examined.

BRASIL. **Pará**: Parauapebas, Flona de Carajás, Cave N1-15 (06°02'03"S, 50°16'17"W), Magangá, 2♀ 1imm., 28/IX-30/X/2007, R. Andrade col. (IBSP 97871); Cave N1-37 (06°01'51"S, 50°16'29"W), Bial, 1♀ 2imm., 28/IX-30/X/2007, R. Andrade col. (IBSP 97870); Cave N4E-08 (06°02'20"S, 50°09'36"W), 2 imm., 20/IV-04/V/2010, C.A.R. Souza col. (IBSP 191216); Cave N4E-15 (06°02'09"S, 50°09'35"W), 1♂ 1♀ 9imm., 20/IV-04/V/2010, D. B. Pedroso, D. Bebiano & I. Cizauskas col. (IBSP 191217-191220); Cave N4E-18 (06°02'02"S, 50°10'03"W), 2imm., 20/IV-04/V/2010, J. Mascarenhas (IBSP 191221); Cave N4E-22 (06°02'14"S, 50°10'02"W), 3♀ 4 imm., 20/X-04/V/10, R. Andrade, C.A.R. Souza & D. B. Pedroso (IBSP 97834; IBSP 191222-191223); Cave N4E-26 (06°02'24"S, 50°09'39"W), 1 imm., 18/VIII-03/IX/2009, I. Cizauskas (IBSP 191224); Cave N4E-31 (06°02'26"S, 50°09'36"W), 1 imm., 18/VIII-03/IX/2009, D. Bebiano (IBSP 191225); Cave N4E-33 (06°01'58"S, 50°09'38"W), 1♂ 1♀, 15-22/IX/2009, I. Cizauskas (IBSP 191226); Cave N4E-33 (06°01'58"S, 50°09'38"W), 3 imm., 15-22/IX/2009, D. B. Pedroso & I. Cizauskas (IBSP 191227-191228); Cave N4E-39 (06°02'22"S, 50°09'38"W), 1 imm., 19/II-04/III/2010, D. B. Pedroso (IBSP 191229); Cave N4E-51 (06°02'00"S, 50°09'12"W), 1 imm., 19/II-04/III/2010, D. Mello (IBSP 191230); Cave N4E-62 (06°01'57"S, 50°09'04"W), 2♀ 1imm., 19/II-04/III/2010, D. B. Pedroso & J. Mascarenhas (IBSP 191231-191232); Cave N4E-65 (06°01'54"S, 50°09'02"W), 1 imm., 24-30/VII/2009, D. Mello (IBSP 191233); Cave N4E-68 (06°01'56"S, 50°09'13"W), 1 imm., 19/II-04/III/2010, C.A.R. Souza (IBSP 191234); Cave N4E-73 (06°01'58"S, 50°09'20"W), 1♂ 2♀ 2 imm., 24-30/VII/2009-04/III/2010, D. Bebiano, J.B. Verdiani & R. Andrade (IBSP 191235-191237); Cave N4E-74 (06°01'57"S, 50°09'02"W), 1imm., 19/II-04/III/2010, I. Cizauskas (IBSP 191238); Cave N4E-77 (06°01'58"S, 50°09'04"W), 11 imm., 24/VII/2009-04/III/2010, I. Cizauskas, D. B. Pedroso & J. Mascarenhas (IBSP 191239-191242); Cave N4E-80 (06°01'58"S, 50°09'21"W), 2 imm., 24/VII/2009-04/III/2010, I. Cizauskas & D. B. Pedroso (IBSP 191243-191244); Cave N4E-82 (06°02'00"S, 50°09'13"W), 1♀, 24-30/VII/2009, D. B. Pedroso (IBSP 191245); Cave N4E-84 (06°02'05"S, 50°09'37"W), 1 imm., 24-30/VII/2009, R. Andrade (IBSP 191246); Cave N4WS-04 (06°04'21"S, 50°11'42"W), 1 imm., 18/XI-01/XII/2010, L. Tunes col. (IBSP 191247); Cave N4WS-04 (06°04'21"S, 50°11'42"W), 2 imm., 10-19/V/2011, I. Cizauskas (IBSP 191248-191249); Cave N4WS-15 (06°03'57"S, 50°11'20"W), 2 imm., 20/IV-04/V/2010, C.A.R. Souza & D.B. Pedroso (IBSP 191250-191251); Cave N4WS-18 (06°04'01"S, 50°11'37"W), 1 imm., 18/XI-01/XII/2010, C.A.R. Souza (IBSP 191252); Cave N4WS-47 (06°04'34"S, 50°11'39"W), 1 imm., 18/XI-01/XII/2010, C.A.R. Souza (IBSP 191253); Cave N4WS-67 (06°04'21"S, 50°11'29"W), 1♀ 14 imm., 18/XI/2010-19/V/2011, V. Felice et al. (IBSP 191254-191259); Cave N4WS-73 (06°04'24"S, 50°11'37"W), 2 imm., 18/XI-01/XII/2010, V. Felice & C.A.R. Souza (IBSP 191260-191261); Cave N5S-03 (06°06'18"S, 50°08'04"W), 2♀, 14-23/X/2009, I. Cizauskas (IBSP 161138); Cave N5S-04 (06°06'19"S, 50°08'02"W), 1♂ 3♀ 10 imm., 14-23/X/2009, D.B. Pedroso & I. Cizauskas (IBSP 161140, IBSP 161189, IBSP 161191); Cave N5S-07 (06°06'20"S, 50°07'59"W), 1♀ 9 imm., 14-23/X/2009, I. Cizauskas, D.B. Pedroso & J.B. Verdiani (IBSP 161158, IBSP 161161, IBSP 161179, IBSP161182); Cave N5S-08 (06°06'20"S, 50°07'56"W), 4♂ 16♀ 49 imm., 7/X/2008-23/X/2009, R. Andrade, I. Cizauskas & J.B. Verdiani (IBSP 191262-191265, IBSP 161106, IBSP 161120, IBSP 161130, IBSP 161141, IBSP 161149, IBSP 161166, IBSP 161184; 1♂ 1♀ for SEM); Cave N5S-09 (06°06'21"S, 50°07'52"W), 1♂ 13♀ 14 imm., 14-23/X/2009, D. B. Pedroso, I. Cizauskas & J.B. Verdiani (IBSP 161108, IBSP 161127, IBSP 161133, IBSP 161215, IBSP 161226 IBSP 161231, IBSP 161233, IBSP 191266); Cave N5S-10 (06°06'20"S, 50°07'53"W), 9♀ 11 imm., 7/X/2008-23/X/2009, R. Andrade et al. (IBSP 161114, IBSP 161116, IBSP 161148, IBSP 161152, IBSP 161209-161210, IBSP 191267-191268); Cave N5S-11 (06°06'17"S, 50°07'46"W), 5♀, 14-23/X/2009, D. B. Pedroso (IBSP 161175); Cave N5S-13 (06°06'19"S, 50°08'01"W), 2♀ 5 imm., 14-23/X/2009, D. B. Pedroso & I. Cizauskas (IBSP 161113, IBSP 161154); Cave N5S-14 (06°06'19"S, 50°08'00"W), 2♂ 8♀ 12 imm., 14-23/X/2009, D. B. Pedroso & I. Cizauskas (IBSP 161132, IBSP 161203, IBSP 161218, IBSP 191269); Cave N5S-20 (06°05'15"S, 50°07'35"W), 2 imm., 25/VIII-03/IX/2009, I. Cizauskas & J.B. Verdiani (IBSP 161206, IBSP 161208); Cave N5S-21 (06°05'15"S, 50°07'33"W), 10♀ 16 imm., 7-12/X/2008, R. Andrade et al. (IBSP 161104, IBSP 161121, IBSP 161123, IBSP 161128, IBSP 161112, IBSP 160160 , IBSP 161170, IBSP 161174, IBSP 161216, IBSP 161169, IBSP 161194, IBSP 161197, IBSP 161207, IBSP 161234, IBSP 191270); Cave N5S-22 (06°05'15"S, 50°07'32"W), 2 imm., 25/VIII-03/IX/2009, I. Cizauskas & J. Mascarenhas (IBSP 161159, IBSP 161236); Cave N5S-26 (06°05'14"S, 50°07'37"W), 2 imm., 10-19/V/2011, D. Bebiano (IBSP 191271); Cave N5S-30 (06°05'18"S, 50°07'11"W), 1♀ 19 imm., 14/XII/2010- 19/V/2011, I. Cizauskas et al. (IBSP 191272-191280); Cave N5S-31 (06°05'28"S, 50°07'08"W), 1imm., 10-19/V/2011, J. Mascarenhas (IBSP 191281); Cave N5S-37 (06°06'21"S, 50°07'56"W), 14♂ 33♀ 86 imm., 7/X/2008-04/IV/2010, J. Mascarenhas et al (IBSP 126125, IBSP 161105, IBSP 161110- 161111, IBSP 161115, IBSP 161124, IBSP 161126, IBSP 161131, IBSP 161134, IBSP 161139, IBSP 161145-161146, IBSP 161164, IBSP 161176- 161178; IBSP 161185-161186, IBSP 161190, IBSP 161192, IBSP 161200, IBSP 161211, IBSP 161213- 161214, IBSP 161221-161222, IBSP 161224-161225, IBSP 161237, IBSP 191282-191283, 2♂ for SEM/MPEG); Cave N5S-38 (06°06'21"S, 50°07'59"W), 1 imm., 14/III-04/IV/2010, C.A.R. Souza (IBSP 161181); Cave N5S-42 (06°06'21"S, 50°08'02"W), 3 imm., 25/VIII-03/IX/2009, D. Mello (IBSP 161109); Cave N5S-43 (06°06'21"S, 50°08'00"W), 1 imm., 14/III-04/IV/2010, J. Mascarenhas (IBSP 161117); Cave N5S-52/53 (06°06'27"S, 50°07'59"W), 1♂ 11♀ 24 imm., 25/VIII/2009-04/IV/2010, J.B. Verdiani et al. (IBSP 16111, IBSP 161118 -161119, IBSP 161143, IBSP 161168, IBSP 161198, IBSP 161219- 161220, IBSP 161227, IBSP 161232); Cave N5S-54 (06°06'28"S, 50°07'59"W), 1 imm., 25/VIII-03/IX/2009, D . Bebiano (IBSP 191284); Cave N5S-55 (06°06'29"S, 50°07'56"W), 2♂ 6♀ 22 imm., 25/VIII/2009-14/IV/2010, D. B. Pedroso et al. (IBSP 161122, IBSP 161155, IBSP 161163, IBSP 161201, IBSP 161212, IBSP 161238 – photography, IBSP 191285 epigynum drawed; IBSP 161403, MEV, internal epigynum); Cave N5S-56 (06°06'27"S, 50°07'57"W), 2 imm., 25/VIII-03/IX/2009, D. B. Pedroso (IBSP 191286); Cave N5S-62 (06°06'17"S, 50°08'06"W), 11 imm., 15/IX/2009-04/IV/2010, D. B. Pedroso & J. Mascarenhas (IBSP 161150, IBSP 161204, IBSP 191288); Cave N5S-63/64/65 (06°06'12"S, 50°08'07"W), 1♂ 5♀ 8 imm., 15/IX/2009-04/IV/2010, D.B. Pedroso, I. Cizauskas & J. Mascarenhas (IBSP 161127, IBSP 161135, IBSP 161144, IBSP 161157, IBSP 161165, IBSP 161195); Cave N5S-66 (06°06'11"S, 50°08'07"W), 1 imm., 15-21/IX/2009, C.A.R. Souza (IBSP 191289); Cave N5S-70 (06°06'05"S, 50°08'03"W), 1♂ 2♀ 8 imm., 25/VIII/2009-04/IV/2010, I. Cizauskas & D. B. Pedroso (IBSP 161129-161230, IBSP 161156, IBSP 161196, IBSP 161205); Cave N5S-74 (06°06'01"S, 50°08'05"W), 3♀ 6 imm., 25/VIII/2009-14/III/2010, D. Mello, D. Bebiano, J.B. Verdiani & C.A.R.de Souza (IBSP 161107, IBSP 161142, IBSP 161151, IBSP 161171, IBSP 161187, IBSP 161228, IBSP 161235); Cave N5S-75 (06°06'02"S, 50°08'01"W), 1♀ 6 imm., 25/VIII/2009-04/IV/2010, R. de Andrade , C.A.R.de Souza & I. Cizauskas (IBSP 161137, IBSP 161162, IBSP 161167, IBSP 161172, IBSP 161183); Cave N5S-79 (06°06'09"S, 50°08'13"W), 4 imm., 15/IX/2009-14/III/2010, D. B. Pedroso & I. Cizauskas (IBSP 161153, IBSP 161199, IBSP 161223); Cave N5S-85 (06°05'11"S, 50°07'34"W), 1♀ 1 imm., 14/III-04/IV/2010, A.R.de Souza (IBSP 161173, IBSP 161229); Cave N5SM1- 031 (06°06'19"S, 50°08'18"W), 1♂ 1♀ 1imm., VIII/2010, M.P. A. Oliveira et al. (ISLA 3935); Cave N5SM2-021 (06°07'58"S, 50°07'51"W), 2♀ 3 imm., 27/X/2010, R. Zampaulo (IBSP 191290); Cave N5SM2-023 (06°08'06"S, 50°08'05"W), 2♀, 11/IV/2011, R. Zampaulo (IBSP 191291); Cave N5SM2-037 (06°07'58"S, 50°08'05"W), 1♂ 2imm., 19/X/2010, R. Zampaulo (IBSP 191292); Cave N5SM2_0001 (06°08'32"S, 50°08'01"W), 1 imm. (ISLA 12360); Cave N5SM2_0006 (06°08'27"S, 50°08'09"W), 1 imm. (ISLA 12370); Cave N5SM2_0016 (06°08'17"S, 50°07'59"W), 2♀ 8 imm. (ISLA 12357; ISLA 12382); Cave N5SM2_0023 (06°08'06"S, 50°08'05"W), 4♀ (ISLA 12378); Cave N5SM2_0026 (06°08'09"S, 50°08'06"W), 3♀ 7 imm. (ISLA 12354; ISLA 12362; ISLA 12377); Cave N5SM2_0027 (06°08'06"S, 50°08'12"W), 1 imm. (ISLA 12351); Cave N5SM2_0033 (06°08'02"S, 50°08'08"W), 1 imm. (ISLA 12350); Cave N5SM2_0037 (06°07'58"S, 50°08'05"W), 2♀ 4 imm. (ISLA 12366; ISLA 12385); Cave N5SM2_0039 (06°07'58"S, 50°08'06"W), 2 imm. (ISLA 12349; ISLA 12375); Cave N5SM2_0040 (06°07'58"S, 50°08'11"W), 11 imm. (ISLA 12347; ISLA 12383); Cave N5SM2_0041 (06°07'58"S, 50°08'12"W), 3 imm. (ISLA 12364; ISLA 12384); Cave N5SM2_0042 (06°07'57"S, 50°08'11"W), 3♀ 7 imm. (ISLA 12355; ISLA 12381); Cave N5SM2_0043 (06°07'56"S, 50°08'10"W), 1♀ 3imm. (ISLA 12358; ISLA 12388); Cave N5SM2_0045 (06°07'54"S, 50°08'05"W), 4♀ 11 imm. (ISLA 12365; ISLA 12387); Cave N5SM2_0046 (06°07'54"S, 50°08'06"W), 1 imm. (ISLA 12363); Cave N5SM2_0049 (06°07'52"S, 50°08'05"W), 2♀ 17 imm. (ISLA 12361; ISLA 12379); Cave N5SM2_0054 (06°07'48"S, 50°08'04"W), 1 imm. (ISLA 12368); Cave N5SM2_0057 (06°07'47"S, 50°08'05"W), 1 imm. (ISLA 12373), Cave N5SM2_0058 (06°07'46"S, 50°08'05"W), 2♀ 5 imm. (ISLA 12352; ISLA 12367); Cave N5SM2_0065 (06°07'41"S, 50°08'08"W), 3 imm. (ISLA 12348); Cave N5SM2_0076 (06°07'31"S, 50°07'54"W), 1 imm. (ISLA 12376); Cave N5SM2_0078 (06°07'23"S, 50°07'48"W), 3♀ 15 imm. (ISLA 12353; ISLA 12386); CaveN5SM2_0086 (06°07'16"S, 50°07'47"W), 3 imm. (ISLA 12356); Cave N5SM2_0089 (06°07'15"S, 50°07'44"W), 1 imm. (ISLA 12369), Cave N5SM2_0092 (06°07'17"S, 50°07'55"W), 1 imm. (ISLA 12372); Cave N5SM2_0095 (06°07'06"S, 50°07'54"W), 1♀ (ISLA 12380); Cave N5SM2_0098 (06°08'27"S, 50°08'03"W), 1 imm. (ISLA 12359; ISLA 12374); Cave N5SM2_0102 (06°07'17"S, 50°07'52"W), 3 imm. (ISLA 12371), all collected between 2014-2015 by Equipe UFLA; CaveN3_0047 (06°02'27"S, 50°13'40"W, 1 imm., 02-23/VIII/2013 (IBSP 186208); Cave N3_0076 (06°02'28"S, 50°13'36"W, 3 imm., 02-23/VIII/2013 (IBSP 186209, IBSP 186210); Cave N5W_0001 (06°04'47"S, 50°08'W), 1♂ 1 imm., 02-23/VIII/2013 (IBSP 186211); Cave N5W_0003 (06°04'53"S, 50°08'04"W), 2 imm., 04-07/XII/2013 (IBSP 186212; IBSP 186213); Cave N1_0015 (06°02'03"S, 50°16'17"W), 1 ♀ 7 imm., 11/VI-02/VII/2014 (IBSP 186214-IBSP 186218); Cave N1_0060 (06°01'12"S, 50°16'41"W), 1imm., 11/VI-02/VII/2014 (IBSP 186219); Cave N1_0064 (06°01'07"S, 50°16'45"W), 1 imm., 11/VI-02/VII/2014 (IBSP 186220); Cave N1_0156 (06°02'41"S, 50°16'22"W), 1imm., 11/VI-02/VII/2014 (IBSP 186221); Cave N1_0247 (06°01'14"S, 50°16'23"W), 1 imm., 11/VI-02/VII/2014 (IBSP 186222); Cave N1_0073 (06°01'13"S, 50°17'17"W), 1 imm., 16/VII-06/VIII/2014 (IBSP 186223); Cave N1_0170 (06°01'23"S, 50°17'58"W), 1 imm., 16/VII-06/VIII/2014 (IBSP 186224); Cave N8_0038 (06°10'24"S, 50°08'49"W), 1 imm., 16/VII-06/VIII/2014 (IBSP 186225); Cave N1_0025 (06°01'49"S, 50°16'20"W), 1 imm., 04/IX-06/X/2014 (IBSP 186226); Cave N1_0037 (06°01'51"S, 50°16'29"W), 2 imm., 04/IX-06/X/2014 (IBSP 186227; IBSP 186228); Cave N1_0101 (06°01'09"S, 50°16'46"W), 1 imm., 24/II-13/III/2015 (IBSP 186229); Cave N8_0004 (06°10'06"S, 50°09'27"W), 1 imm., 24/II-13/III/2015 (IBSP 186230); Cave N1_0119 (06°01'16"S, 50°18'06"W), 1 imm., 02-29/IV/2015 (IBSP 186231), Cave N8_0008 (06°10'05"S, 50°09'32"W), 1 imm., 02-29/IV/2015 (IBSP 186232), all collected by Equipe Carste.

#### Distribution.

Known only from caves in the state of Pará, northern Brazil (Fig. 19).

#### Natural history.

All 767 specimens (34♂, 179♀, 554 immatures) of *Carajas paraua* sp. n. were collected in 104 caves distributed in rock outcrops covered by canga vegetation (details in Mota et al. 2015) surrounded by the Amazon Forest. This species is restricted to the underground environment of iron caves that exist in canga, in the ore (a rock with more than 60% iron and little or no silica, carbonates, or sulfides) and iron formations (designated for itabirites, ferruginous dolomites, hematitic phyllites, jaspilitos, and hematite) from Flona of Carajás. The specimens were found on the ground, under rocks in aphotic zones with high relative humidity (≥ 98%) and frequently in caves with bat colonies. The tiny size of the fangs of this species seems to indicate that their diet is based on micro invertebrates such as mites, Collembola and Diptera larvae, often found in high abundance in soil areas with bat guano. *Carajas paraua* sp. n. is a troglobite spider with a distribution restricted to iron caves in target areas of mining and regions of iron formation with high economic interest.

## Discussion

The phylogenetic relationships of Caponiidae are unknown, although some studies have inferred the relationship of certain genera of the family (Platnick 1993; Platnick 1994a, b; Platnick and Jager 2008; Platnick and Lise 2007). A study including all genera of the family is being prepared by the authors, and the discussion here is premature.

Among the genera described here, the most remarkable is *Nasutonops* gen. n. All species have a distally projected clypeal horn, not found in other Caponiidae. Despite this interesting modification, the ocular area and genital structures link this species to *Caponina* (see Platnick 1994a). The disposition of the six eyes are similar in both genera, with the median eyes larger than the others (see Platnick 1994a: fig. 19); however, no *Nasutonops* species have an ocular reduction as do some *Caponina* species. The configuration of the male palp is also similar (compare Fig. 11C with Platnick 1994a: fig. 26), with the differences in the position and base of the embolus, which has a flattened base and protrudes distally in *Nasutonops* species (Fig. 11C–E) rather than protruding medially as in *Caponina* (Platnick 1994a, fig. 27). Whether the embolus protrudes from the bulb distally or medially could be a synapomorphy for each genus. The female internal genitalia of *Caponina* species (see Platnick 1994a, figs 22–25; Brescovit and Sánchez-Ruiz 2013, fig. 10) resembles the genitalia of *Nasutonops* species, but species of *Caponina* have a distinctive pair of curved sclerotizations arising from the posterior wall of the bursa copulatrix (see Platnick 1994a, figs 22–25) and is considered a synapomorphy of the genus. Externally, the genitalia are quite different: *Nasutonops* species have a strongly sclerotized trasverse internal fold (absent in *Caponina*), which can be observed externally via a transparent area. This transverse fold runs along nearly the entire epigastric area, reaching the posterior ends of the sclerotized bars in the internal genitalia, and in *Nasutonops sincora* is very wide. The clypeal horn, the distal origin of the embolic base in the male palps, and the sclerotized trasverse internal fold of the female internal genitalia may be synapomorphies supporting the monophyly of *Nasutonops*.

**Figure 18.**
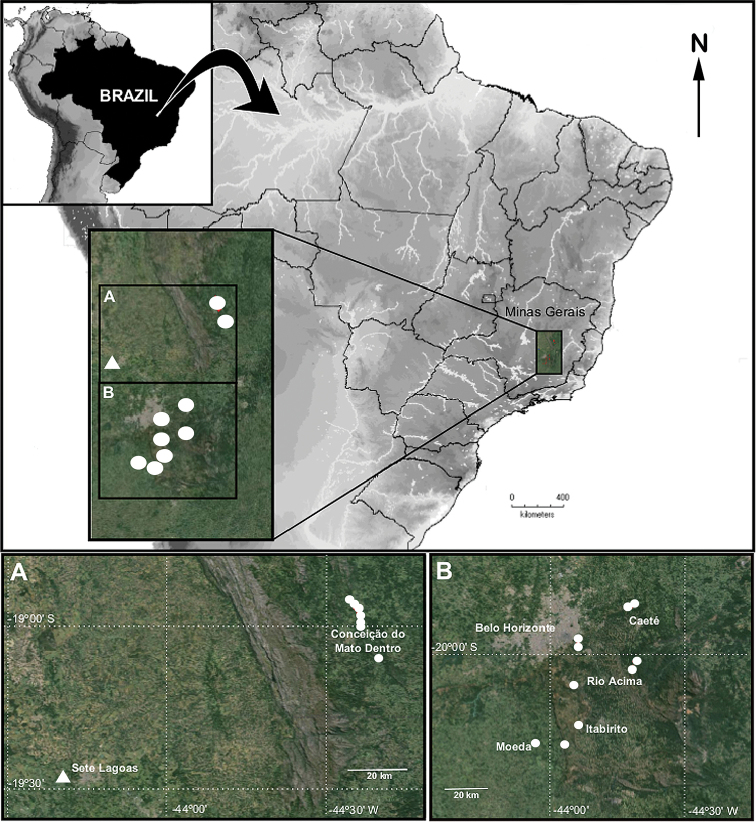
Distribution maps of *Tisentnops mineiro* sp. n (white circles) and *Tisentnops onix* sp. n. (white triangles) from the state of Minas Gerais, Brazil.

**Figure 19.**
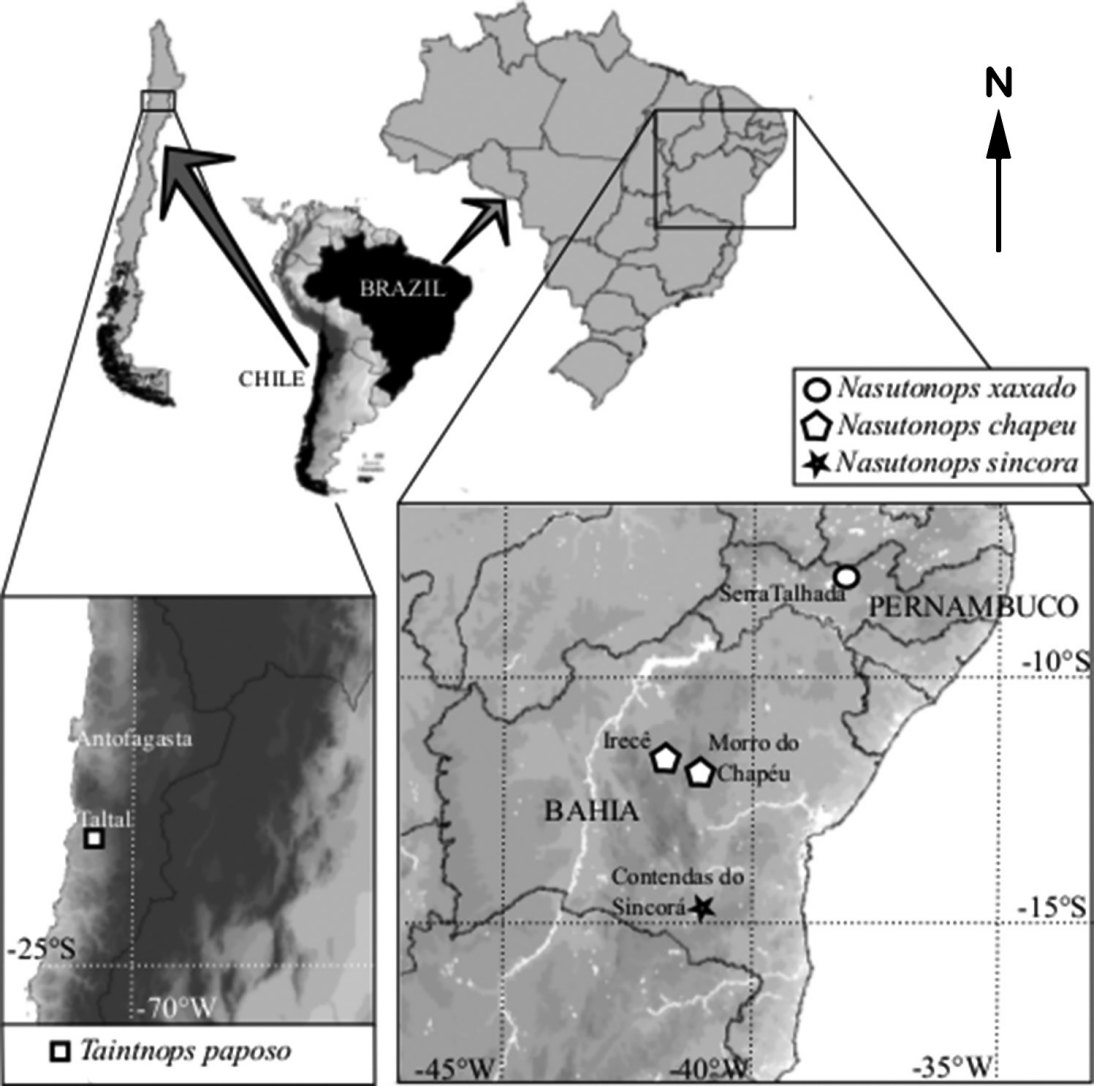
Distribution maps of *Nasutonops* species from the states of Bahia and Pernambuco, Brazil, and *Taintnops paposo* sp. n. from Chile.

The first blind caponiids described here are also remarkable (*Tisentnops mineiro* sp. n. and *Carajas paraua* sp. n.). They were found only in caves and are totally eyeless. Furthermore, they have other troglobitic adaptations, such as very long trichobothria (Figs 3A, 13C, D; 14H) and some modifications to the tarsal claws. Particularly, *Carajas paraua* sp. n. lacks unpaired claws on all tarsi (Figs 14G; 15G, H), and the paired claws on the posterior tarsi have the distal tip thickened and covered with dense and short bristles (Figs 14G, 15H). These dramatic modifications of the tarsal claws, as well as the anteriorly and posteriorly strongly projected endites (Figs 13J, 14D), are unique among caponiids, and may also be related to the cave environment. Specimens of *Carajas paraua* sp. n. and *Tisentnops mineiro* sp. n. were extensively collected in caves from the Brazilian states of Pará and Minas Gerais respectively (Figs 18–19), but were never found outside these caves, where only specimens of *Nops* MacLeay were collected. The other new *Tisentnops* described here (*Tisentnops onix* sp. n.) was also found in a cave, and although it is not eyeless, it has a much reduced single pair of eyes almost on the front of carapace (Fig. 5D–E) and similar distinct trichobothria (Fig. 5H). Diagnostic characters of the genus, such as the uniquely modified endites, and the elongate raised sockets of setae on endites and anterior legs, could be other troglobitic modifications, suggesting that representatives of Brazilian *Tisentnops* apparently live only in caves. However, when Platnick (1994b) redescribed the type species *Tisentnops leopoldi* (Zapfe), he mentioned that a major collection effort to obtain additional material was made without success on two separate occasions at the type and nearby localities from Chile; however, there are no caves in these areas. Therefore, it is unlikely that the Chilean type species is a troglobite.

The description of these two new *Tisentnops* species allowed us to increase the knowledge of this genus. Specifically, the configuration of the female internal genitalia was studied for the first time. The female genitalia of *Tisentnops* resembles that found in *Calponia*, *Carajas* gen. n., *Diploglena*, *Notnops*, *Laoponia* and *Iraponia*, with an elongate membranous anteromedian receptaculum formed by an anteriorly directed median tubular duct leading to a globose sac (see Figs 3G, 15K; Platnick 1993: fig 17; Platnick 1994b: figs 20–21; Platnick and Jager 2008: figs 31–34; Kranz-Baltensperger et al. 2009: figs 47–48; Haddad 2015: fig. 24). The main differences among all genera are in the presence or absence, position and shape of the sclerotized structures forming the transverse bars and folds. Interestingly, *Taintnops* species also have a large, oval anteromedian receptaculum (see Fig. 4H and Platnick 1994b: fig. 25), but lack the anteriorly directed median duct. *Caponina* and *Nasutonops* gen. n. apparently form a separate group with a wide and short uterus externus that is not globose and lacks a median duct; however, they retain the pair of elongate sclerotized bars, which are covered by a transparent hyaline membrane (Fig. 4D–E).

In summary, the female internal genitalia of non-nopine genera may indicate a monophyletic group formed by those genera with a median duct and a globose sac on the membranous anteromedian receptaculum. In this case, *Diploglena* and *Tisentnops* belong to this group and would be closely related as suggested by Platnick and Jager (2008) due to the anteriorly expanded palpal endites. Unfortunately, the male palp appears to be uniform among the non-nopine genera, having a globose bulb with a tubular or lamelliform embolus (Figs 1E–F, 9J). The exception occurs in the genera *Caponia* and *Diploglena* whose bulbs have a tegular apophysis and a membranous conductor (see Purcell 1904: figs 28–35; Haddad 2015: figs 46–47, 64–66).

**Figure 20.**
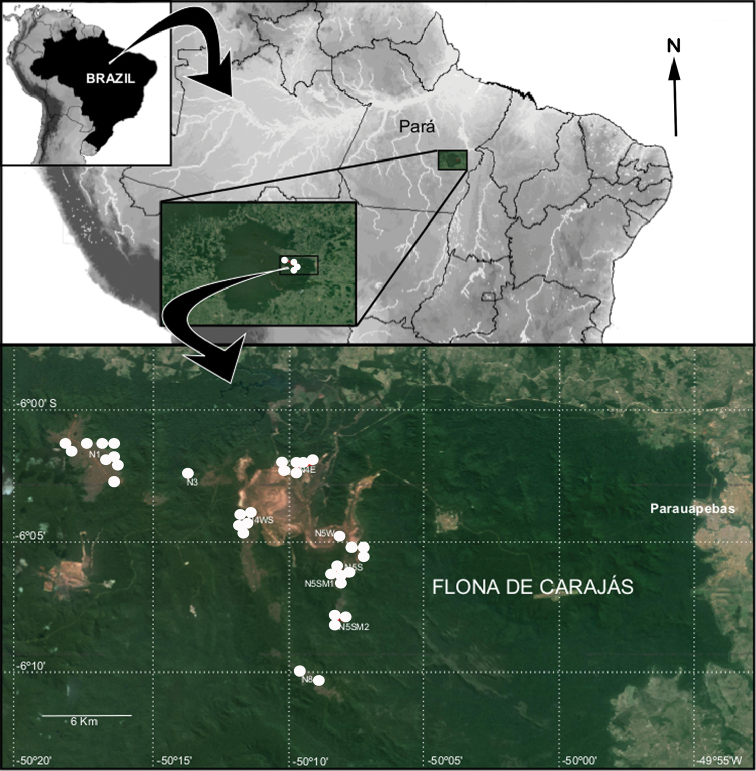
Distribution map of *Carajas paraua* sp. n. (white circles) from the Carajás area, northern Brazil.

## Supplementary Material

XML Treatment for Tisentnops

XML Treatment for Tisentnops mineiro

XML Treatment for Tisentnops onix

XML Treatment for Taintnops

XML Treatment for Taintnops paposo

XML Treatment for Nasutonops

XML Treatment for Nasutonops xaxado

XML Treatment for Nasutonops chapeu

XML Treatment for Nasutonops sincora

XML Treatment for Carajas

XML Treatment for Carajas paraua
